# Unsupervised learning for robust working memory

**DOI:** 10.1371/journal.pcbi.1009083

**Published:** 2022-05-02

**Authors:** Jintao Gu, Sukbin Lim

**Affiliations:** 1 Neural Science, New York University Shanghai, Shanghai, China; 2 NYU-ECNU Institute of Brain and Cognitive Science at NYU Shanghai, Shanghai, China; Research Center Jülich, GERMANY

## Abstract

Working memory is a core component of critical cognitive functions such as planning and decision-making. Persistent activity that lasts long after the stimulus offset has been considered a neural substrate for working memory. Attractor dynamics based on network interactions can successfully reproduce such persistent activity. However, it requires a fine-tuning of network connectivity, in particular, to form continuous attractors which were suggested for encoding continuous signals in working memory. Here, we investigate whether a specific form of synaptic plasticity rules can mitigate such tuning problems in two representative working memory models, namely, rate-coded and location-coded persistent activity. We consider two prominent types of plasticity rules, differential plasticity correcting the rapid activity changes and homeostatic plasticity regularizing the long-term average of activity, both of which have been proposed to fine-tune the weights in an unsupervised manner. Consistent with the findings of previous works, differential plasticity alone was enough to recover a graded-level persistent activity after perturbations in the connectivity. For the location-coded memory, differential plasticity could also recover persistent activity. However, its pattern can be irregular for different stimulus locations under slow learning speed or large perturbation in the connectivity. On the other hand, homeostatic plasticity shows a robust recovery of smooth spatial patterns under particular types of synaptic perturbations, such as perturbations in incoming synapses onto the entire or local populations. However, homeostatic plasticity was not effective against perturbations in outgoing synapses from local populations. Instead, combining it with differential plasticity recovers location-coded persistent activity for a broader range of perturbations, suggesting compensation between two plasticity rules.

## Author summary

While external error and reward signals are essential for supervised and reinforcement learning, they are not always available. For example, when an animal holds a piece of information in mind for a short delay period in the absence of the original stimulus, it cannot generate an error signal by comparing its memory representation with the stimulus. Thus, it might be helpful to utilize an internal signal to guide learning that can make a system resilient. Here, we investigate the role of such unsupervised plasticity for working memory maintenance, which acts during the delay period without external inputs. We consider two prominent classes of learning rules: differential plasticity that corrects the rapid changes of instant neural activity and homeostatic plasticity that regularizes the long-term average of activity. The two learning rules have been proposed to fine-tune the synaptic weights in continuous attractor models suggested for parametric and spatial working memory. Here, we compare their performance under various network perturbations and learning parameters. The systematic studies reveal the conditions under which each rule can effectively stabilize persistent memory and suggest possible synergy between the two learning rules.

## Introduction

Continuous attractors have been hypothesized to support brains’ temporary storage and integration of analog information [1–4]. An attractor is an idealized stable firing pattern that persists in the absence of stimuli. Integration is allowed if these attractors form a continuous manifold. Theoretical models predict that neural activity should be restricted within but free to move along this manifold, making stochastic fluctuation correlated among neurons, as is validated in the brainstem oculomotor neural integrator [5], the entorhinal grid cell system [6], and prefrontal visuospatial selective neurons [7].

Computationally, the performance of continuous attractors is known to be sensitive to network parameters, which is termed as the “fine-tuning problem” [8,9]. A slight imperfection like a synaptic weight asymmetry could make continuous attractors break down into a few discrete attractors or cause an overall drift of activities. This raises the question of how continuous attractors could exist in the brain. Noting that the model is just an idealization, earlier studies have proposed that continuous attractors can be approximated by finely discretized attractors with a hysteresis of coupled bi-stable units, which would make the system more robust [10,11]. Recent theoretical studies suggest other complementary mechanisms, including derivative feedback and short-term facilitation, with the former slowing down activity decay [12,13] and the latter transiently enhancing stability [14,15].

These workarounds could make continuous attractors more tolerant to perturbations in connectivity strengths or heterogeneity of single neuronal properties. Not mutually exclusively, long-term plasticity is believed to take part in settling a reasonable parameter range. For example, the plasticity involved in the fish oculomotor integrator has been most studied. Previous works have proposed either visually supervised plasticity [16–18] or self-monitoring plasticity acting in the dark [19,20]. These plasticity rules utilize time-derivative signals to detect slips in the eye position or changes in neural activity, so-called differential plasticity. Note that similar mechanisms can be generalized to mediate the tuning conditions of the parametric working memory encoding analog information [12,18,21]. More broadly, derivative-based rules have been suggested to learn temporal relationships between input and output [22–24] and in reinforcement learning [25–27].

Another class of long-term synaptic plasticity for stabilizing continuous attractors is homeostatic plasticity, which regularizes the excitability of neurons [28]. Many models focused on the role of homeostatic plasticity to prevent instability. As homeostatic plasticity tends to pull excitation down or boost inhibition when network activity is higher than a reference value, a positive feedback between network activity and activity-dependent plasticity can be counterbalanced [29]. On the other hand, Renart et al. [30] considered network storing spatial information in spatially localized “bump” activity and proposed an additional role of homeostatic plasticity, that is to regularize the network patterns and recover tuning condition for spatial working memory perturbed by the heterogeneity of local excitability. Similarly, Pool and Mato [31] suggested that for developing orientation selectivity through Hebbian learning in recurrent connections, homeostatic plasticity can enforce symmetry in synaptic connections such that all orientations can be represented equally in the networks.

Both differential and homeostatic plasticity suggested for attractor networks are unsupervised. External supervisory or reward signals are not required to achieve the tuning condition to form continuous attractors. As shown previously, they can act after the offset of sensory signals and might be suitable for memory tasks that typically have a long memory period without external input. However, previous works have investigated the effect of differential plasticity and homeostatic plasticity partially for different types of continuous attractor or under particular types of perturbations in the network connectivity or inhomogeneity of neuronal properties.

Therefore, we investigated whether these two forms of learning can stabilize persistent activity in continuous attractors, which require fine-tuning conditions of network parameters. As a systematic study, we considered two different types of continuous attractors, namely, rate-coded and location-coded persistent memory, under which memory neurons show monotonic tuning of an encoded feature or bell-shaped tuning, respectively [2,4]. For both types of memory, we considered a single framework, called the negative derivative feedback mechanisms [12,13]. First, we formally described the fine-tuning problem in a rate-coded attractor system with a simpler network architecture than a location-coded attractor. We examined the effects of differential plasticity and homeostatic plasticity and how recovery from perturbation in connectivity depends on the learning parameters. Then we extended the scope of our investigation to a location-coded system that requires spatially structured networks and investigated the recovery of tuning conditions under various types of perturbations. Finally, we demonstrated that two rules could partially compensate for each other when they are combined.

## Results

### Rate-coded persistent activity in one homogenous population

Before we discuss the synaptic plasticity rule that stabilizes persistent spatial patterns of activity, we first consider the similar mechanism applied for a rate-coded persistent activity where the persistent firing rate of memory neurons varies monotonically with the encoded signals [2]. Compared to location-coded memory suggested for maintaining spatial information, the rate-coded one has been suggested to maintain graded-level information such as somatosensory vibration frequency [32,33]. Previous theoretical works proposed that recurrent circuits can maintain both types of memory based on similar feedback mechanisms despite the different network architecture [13]. Thus, we first gain insight into how the specific form of synaptic plasticity can stabilize persistent memory in the rate coding scheme, which has a simpler network structure.

As the rate-coded network can be built upon a spatially homogeneous structure, its dynamic principle can be captured in the mean-field equations describing the network dynamics with one variable (Methods). Two representative feedback mechanisms can be present based on recurrent network interactions, positive feedback and negative derivative feedback, both of which is described by the following equation,

$$\frac{dr}{dt}=-r+w_{net}r-w_{der}\frac{dr}{dt}+I(t).$$ (1)

In the above equation, *r* represents the mean firing rate of the network activity. We considered that time *t* and other time constants are unitless (normalized with the intrinsic time constant of *r*) for simplicity. The first and last terms on the right side represent the intrinsic leakage and transient external input. The second and third terms represent the feedback arising from recurrent inputs.

In the positive feedback models, the excessive excitatory inputs need to be tuned to cancel the intrinsic leakage such that the net gain *w*_*net*_ in the second term is tuned to be one, whereas *w*_*der*_ is typically zero [12]. On the other hand, in the negative derivative feedback models, balanced excitatory and inhibitory recurrent inputs with different kinetics generate the resistive force against memory slippage, similar to time-derivative activity in the third term (Methods). As its strength represented by *w*_*der*_ increases with overall recurrent synaptic currents while the second term remains relatively small for balanced excitation and inhibition, Eq 1 is approximated by $w_{der}\frac{dr}{dt}=I(t)$. Thus, for large negative derivative feedback, the effective time constant of decay of network activity increases proportionally, and the decay of activity slows down [12]. Note that the feedforward input to be integrated is *I*(*t*)/*w*_*der*_. This ratio can remain constant for large *w*_*der*_ if the feedforward input strengths *I(t)* increase together with the recurrent one as well as *w*_*der*_ as considered in typical balanced network models [12,34].

**Fig 1:**
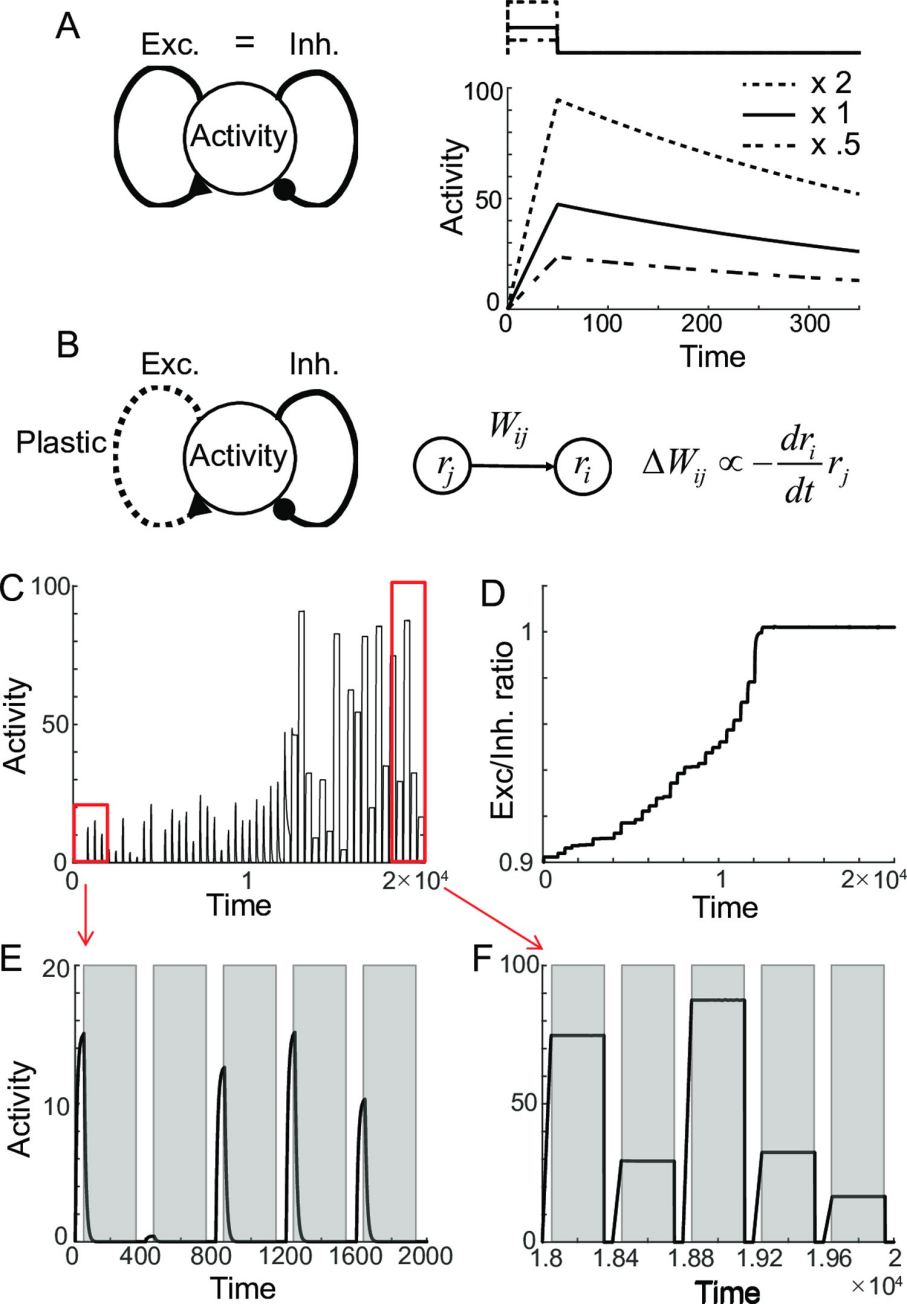
Recovery of rate-coded persistent activity through differential plasticity. A: Maintenance of persistent activity through negative-derivative feedback. With balanced excitation and inhibition as well as slower excitation, the network can maintain persistent activity at different rates. The top panel illustrates the schematic of a pulse-like stimulus. The dotted and dash-dotted curves in both top and bottom panels represent stimulus and activity with double and half the input strengths compared to the solid one. B: Schematics of differential plasticity in the excitatory feedback. C-D: Recovery of persistent activity (C) and E-I balance under differential plasticity (D) after perturbations in connectivity strengths. E-F: Activities with 10% perturbation (E) and after the recovery (F). The time axis is in the unit of intrinsic time constant τ, and one trial is composed of the stimulus presentation, delay period, and an inter-trial interval. Shaded areas represent the delay period during which the plasticity occurs.

With a long effective time-constant of decay, both networks show integrator-like properties such that during the stimulus presentation, it integrates the external input. After its offset, it maintains persistent activity at different levels (Fig 1A). However, any memory circuits keeping the information in continuum states face a fine-tuning problem [8,9,35]. Similarly, for rate-coded persistent memory, despite the different tuning conditions in positive feedback models and negative-derivative feedback models, the deviation from the perfect tuning leads to a gross disruption of persistent activity. For instance, a reduction in the E-to-E connection causes an imbalance between the recurrent excitation and inhibition in negative derivative feedback models and leads to the rapid decay of the activity (Fig 1E). Such an E-to-E perturbation has been suggested to underlie the disruption of persistent firing in memory cells observed experimentally under the application of NMDA blockade [12,36].

### Stabilization of persistence through differential plasticity

To mitigate this fine-tuning condition and to make the network resilient against perturbations, several forms of synaptic plasticity have been proposed. Two prominent synaptic plasticity rules suggested for persistent activities are homeostatic plasticity [28,30] and differential plasticity [19,20]. Here, we examine how each plasticity can stabilize a rate-coded persistent activity.

First, we consider differential synaptic plasticity where the synaptic update depends on the firing rates of pre- and postsynaptic neurons and their time derivatives (Fig 1B; [19]). Previous work showed that such a plasticity rule updates the synaptic connections to reduce the overall derivative of network activities [19]. We considered the negative-derivative feedback model composed of one homogenous population to understand further how the fine-tuning condition can be achieved through the differential plasticity rule. If initially balanced excitation and inhibition is perturbed by the reduction in the excitatory connection and excitatory connection changes according to the differential plasticity rule, the dynamics of the system can be captured by the firing rate *r* and excitatory connection strength *W*_*exc*_ as

$$\begin{array}{c} \frac{dr}{dt}=-r+(W_{exc}-W_{inh})r-w_{der}\frac{dr}{dt}+I(t)\\ \frac{dW_{exc}}{dt}=-\alpha \frac{dr}{dt}r \end{array}$$ (2)

where *w*_*net*_ is replaced by *W*_*exc*_*−W*_*inh*_ in Eq 1, and *w*_*der*_ is proportional to *W*_*inh*_ multiplied by the difference of the time constants for excitatory and inhibitory inputs feedback (Methods).

The steady states of the system are *r* = 0 or *dr*/*dt* = 0, where the latter can be achieved for balanced excitation and inhibition, that is, *W*_*exc*_/*W*_*inh*_ ~ 1 for large *W*_*inh*_. We simulated the dynamics in successive trials, where each trial is composed of stimulus presentation and delay period followed by the inter-trial interval (Fig 1C). Note that we assume that the plasticity rule modifies the synaptic strengths only during the delay period (shaded area in Fig 1E and 1F; Discussion). That is, during the stimulus presentation, the external input *I*(*t*) is on and plasticity is off, while it is opposite during the delay period such that *I*(*t*) is off and plasticity is on. During the inter-trial interval, the activity is set to zero and plasticity is naturally off.

**Fig 2:**
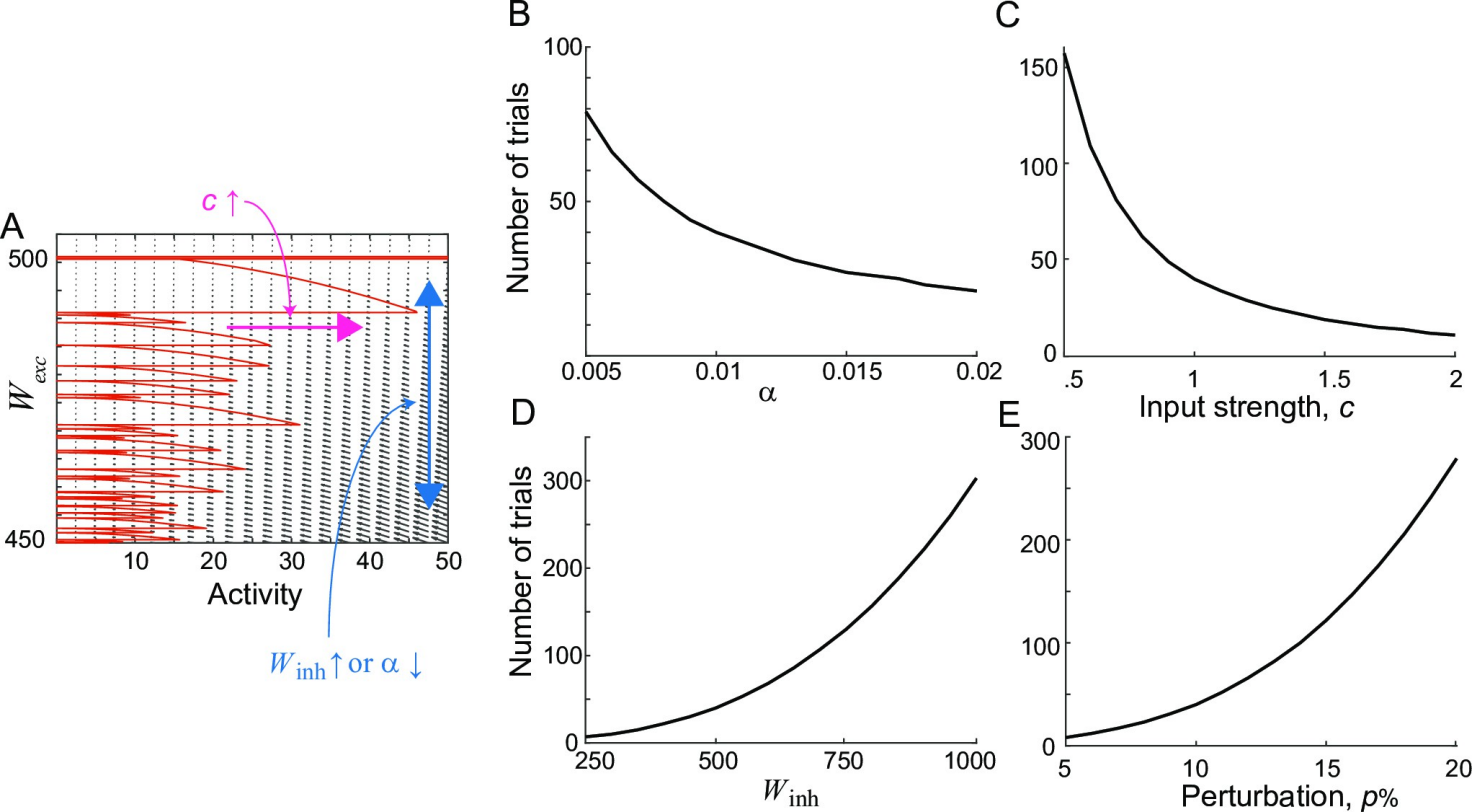
Recovery dynamics dependence on learning parameters under differential plasticity. A: Phase-plane of activity *r* and synaptic strength of recurrent excitation *W*_*exc*_. The small black arrows represent a vector field for the dynamics of *r* and *W*_*exc*_, described in Eq 2. The red curve is a trajectory starting from 10% perturbation in *W*_*exc*_, that is, *W*_*exc*_ = 0.9*W*_*inh*_ with *W*_*inh*_ = 500. During the stimulus presentation, the trajectory jumps horizontally, and input strengths vary randomly across trials. The big arrows indicate the effects of changing the learning speed α or *W*_*inh*_ (blue vertical) and relative mean input strengths *c* (magenta horizontal). B-E: Dependence of recovery speed on learning and network parameters. The minimum number of trials for *W*_*exc*_ to reach up to 0.99*W*_*inh*_, that is, about 1% from perfect tuning was obtained by varying α (B), *c* (C), *W*_*inh*_ (D), perturbation strength *p* (E). All parameters change from 50% to 200% of those used in Fig 1.

In these successive trials, the “*r* = 0” steady state cannot be maintained because *r* is reset to a nonzero value during the stimulus presentation in each trial. In the phase plane of *r* and *W*_*exc*_, the evolution of the system during the delay period corresponds to a smooth trajectory following the vector field defined by Eq 2 with *I*(*t*) = 0. On the other hand, during the stimulus presentation, external input increases *r* without changing *W*_*exc*_, leading to a horizontal jump in the trajectory (Fig 2A). In initial trials, *W*_*exc*_ is deficient compared to *W*_*inh*_, resulting in activity drift (Fig 1C and 1E). The drift drives differential plasticity to potentiate *W*_*exc*_. As a result, *W*_*exc*_ recovers over the trials (Fig 1D and Fig 2A). Once the balanced tuning condition with *W*_*exc*_ ≈ *W*_*inh*_ is achieved, the network can maintain the graded level of persistent activities (Fig 1F).

We further investigated how the speed for recovery of the tuning condition depends on the parameters of differential plasticity (Fig 2). Mathematical analysis revealed the relationship between the effects of changing the learning speed α, *W*_*inh*_, and overall input strengths during the stimulus presentation, denoted as *c*. Increasing *c* is equivalent to increasing α to the second power, while increasing *W*_*inh*_ is equivalent to decreasing α to the third power (Fig 2B, 2C, and 2D; Methods). Intuitively, increasing *W*_*inh*_ is effectively the same as stretching the *W*_*exc*_-axis, resulting in a similar effect to decreasing α (blue vertical arrow in Fig 2A). Thus, stronger derivative feedback with larger *W*_*inh*_ requires a longer time to recover after the same percentage of perturbation (Fig 2D). On the other hand, *c* determines the increment of *r* during the stimulus presentation such that larger stimulus strength pushes the system in a faster speed regime and makes the system converge faster (magenta horizontal arrow in Fig 2A and 2C).

Another parameter is the perturbation strength *p*. Analytically, we found the relationship between α and *p* in a special case–in a single trial with the same initial state of activity, increasing *p* or decreasing α leads to the same final activity if the final state is a balanced one (Methods). While such an analytical derivation holds only for a single trial reaching the balanced state, we found a qualitatively similar inverse relationship in multiple trials. Increasing *p* sets the initial *W*_*exc*_ further away from the final balanced state and results in longer recovery to the balanced state, similar to decreasing α (Fig 2B and 2E)

### Homeostatic plasticity is effective but sensitive

**Fig 3:**
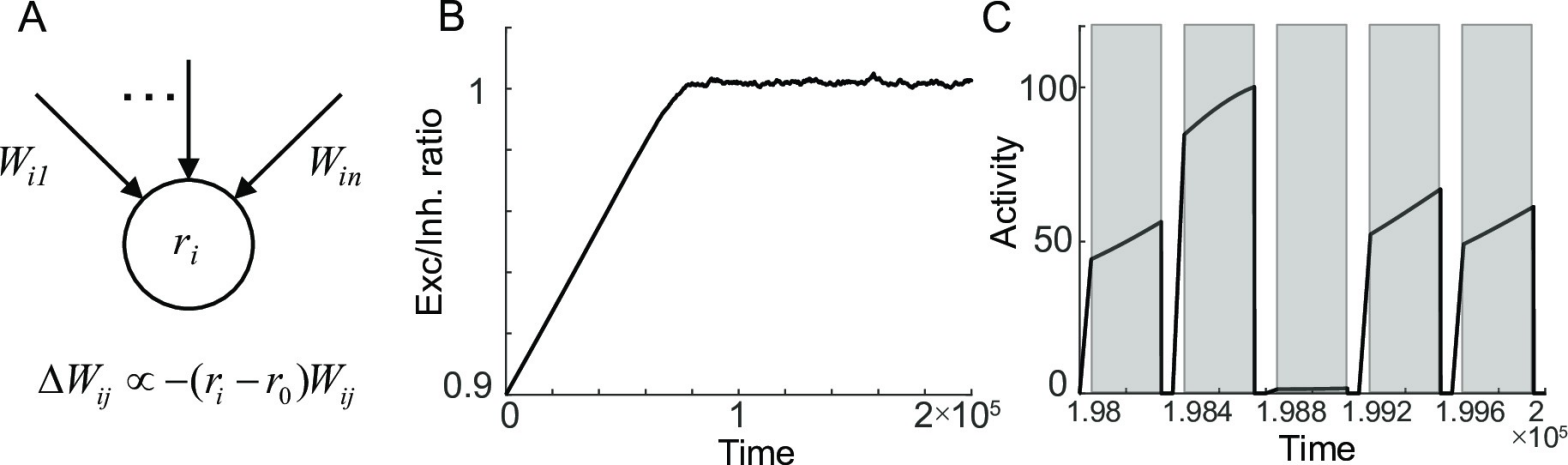
Recovery of rate-coded persistent activity through homeostatic plasticity. A: Schematics of homeostatic plasticity scaling the strengths of incoming synapses to achieve the target firing rate *r*_*0*_. B-C: Recovery of E-I balance after perturbations in connectivity strengths (B) and maintenance of persistent activity at the different levels after the recovery (C).

While differential plasticity has been shown to stabilize the rate-coded persistent activity [12,19,20], homeostatic plasticity has been suggested to stabilize different forms of memory, such as spatial working memory [30] and discrete working memory [37,38]. Homeostatic plasticity regulates the excitability of postsynaptic neurons. In its typical form, all incoming synapses onto the postsynaptic neurons multiplicatively scale for the long-term average rate to achieve their target firing rates *r*_0_ (Fig 3A). As for differential plasticity, we examined the effect of homeostatic plasticity in one homogenous population for a rate-coded persistent activity, whose dynamics is described as

$$\begin{array}{c} \frac{dr}{dt}=-r+(W_{exc}-W_{inh})r-w_{der}\frac{dr}{dt}+I(t)\\ \frac{dW_{exc}}{dt}=-\alpha W_{exc}(r-r_{0}). \end{array}$$ (3)

The steady state of such a system is achieved when *r = r*_*0*_ and *dr*/*dt* = 0, that is, *W*_*exc*_ ≈ *W*_*inh*_ for large *W*_*inh*_. Note that this is more stringent than those for differential plasticity that requires the latter balance condition only.

**Fig 4:**
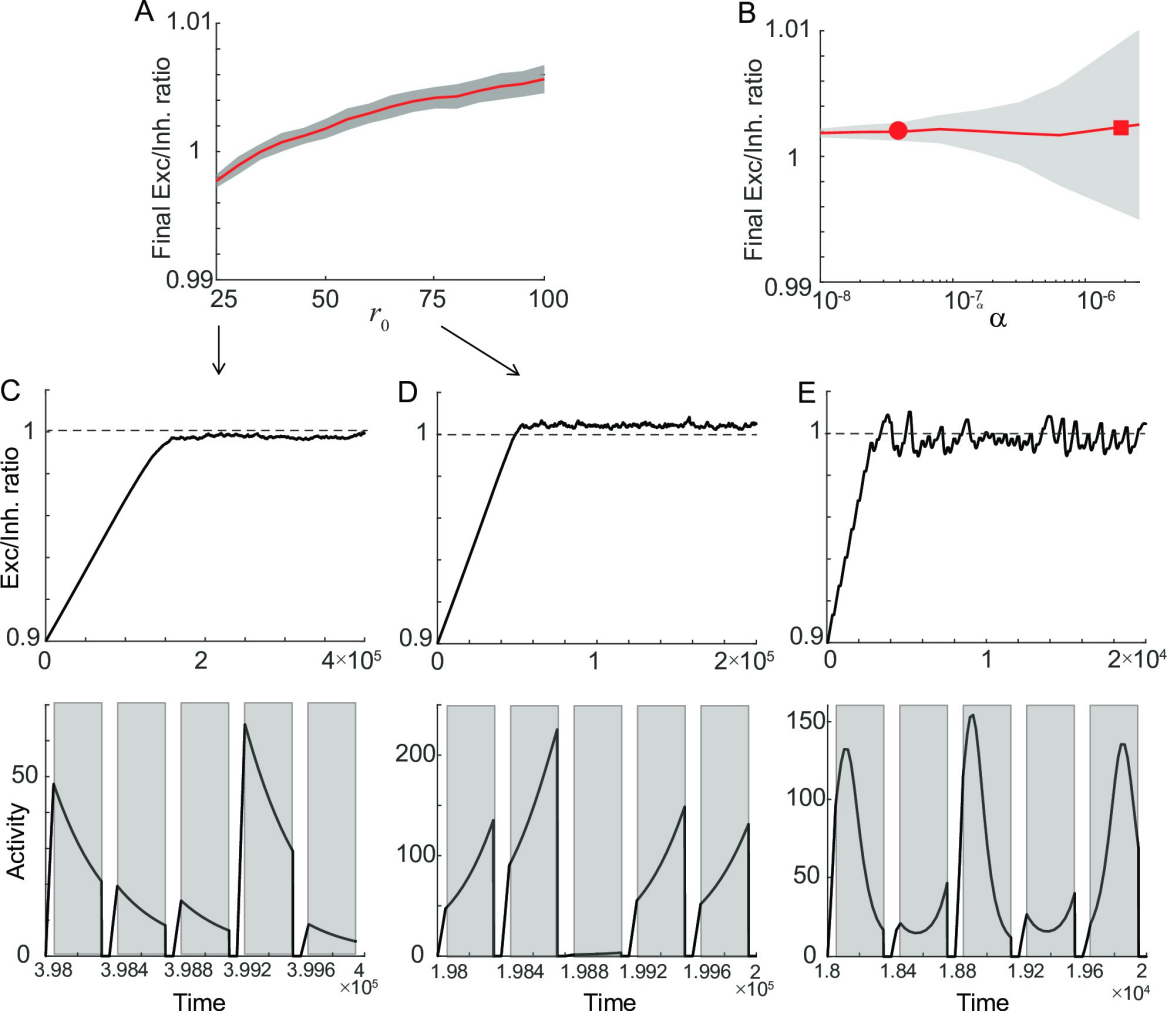
Sensitivity of homeostatic learning rule on learning parameters. A-B: Dependence of final balance ratio *W*_*exc*_/*W*_*inh*_ on *r*_*0*_ (A) and α (B). After reaching the steady state, *W*_*exc*_/*W*_*inh*_ was averaged over the trials whose mean and standard deviation were shown as red curve and graded area. C-D: Evolution of *W*_*exc*_/*W*_*inh*_ over trials (top) and the activity after reaching the steady state (bottom) for lower *r*_*0*_ (C) and higher *r*_*0*_ (D) compared to that in Fig 3B and 3C. E: Sensitivity to learning speed α. For a faster learning rate, the homeostatic plasticity leads to the oscillation even for properly tuned *r*_*0*_, leading to a larger standard deviation (square in B) compared to a slower learning rate (circle in B corresponding to Fig 3B and 3C).

Like differential plasticity, we found that the steady state can be achieved through homeostatic plasticity (Fig 3B). However, it requires additional tuning of parameters such as the target rate *r*_0_, the mean input strength *c*, and inhibitory feedback strength *W*_*inh*_. For instance, given *c* and *W*_*inh*_ determining the value of *r* at the beginning of the delay, *r*_*0*_ should match the mean of initial *r* over trials to achieve the balance condition and stabilize the rate-coded persistent activity (Fig 3B and 3C). However, for inadequately tuned *r*_*0*_, the balanced state cannot be achieved. With decreasing *r*_*0*_, the mean of initial *r* becomes larger than *r*_*0*_, and the dynamics of *r* drifts downward to achieve *r*_*0*_ on average during the delay period (Fig 4A and 4C, bottom). Consequently, *W*_*exc*_ is stabilized to be deficient compared to *W*_*inh*_ (Fig 4C, top), whereas increasing *r*_*0*_ leads to the upward drift of activity and excessive *W*_*exc*_ (Fig 4D). We found that changing *c* or *W*_*inh*_ affects the final *W*_*exc*_/*W*_*inh*_ similarly to changing *r*_*0*_ (S1 Fig and Methods).

We also examined the effect of other parameters, learning speed α and perturbation strength *p*. Unlike *r*_*0*_, *c* or *W*_*inh*_, changing α or *p* does not affect the final balance state but affects the recovery speed (Figs 4B and S1). Increasing α or decreasing *p* reduces the number of trials to reach the balanced *W*_*exc*_/*W*_*inh*_ as in differential plasticity. However, a local stability analysis that utilizes the eigenvalues of the Jacobian matrix at the steady state further reveals that near the steady state, the system shows oscillations whose frequency depends on α such that larger α leads to faster oscillation (Methods). In successive trials with reset in *r*, larger α leads to the ongoing oscillation near the balanced *W*_*exc*_/*W*_*inh*_ even for properly tuned learning parameters (square in Fig 4B and 4E). Overall, the analysis of one homogenous population shows that although homeostatic rule can stabilize persistent activity for rate-coded memory, the balance condition and stability are sensitive to learning parameters.

### Location-coded persistent memory in spatially structured network

So far, we have shown how two prominent plasticity rules can stabilize rate-coded persistent memory in one homogenous population. However, whether the same mechanism can be generalized to stabilize location-coded persistent memory is in question. While rate-coded persistent memory can be encoded in the amplitude of the homogeneous population, it was suggested that location-coded persistent memory is encoded in the spatial pattern of multiple populations connected through distance-dependent weights [2,4]. However, both differential and homeostatic plasticity rules are local, depending on pre- and postsynaptic activities but have no regularization on a spatial pattern of activities. Here, we consider the negative derivative feedback model suggested for spatial working memory [13] and explore under which condition each plasticity can stabilize location-coded persistent memory.

**Fig 5:**
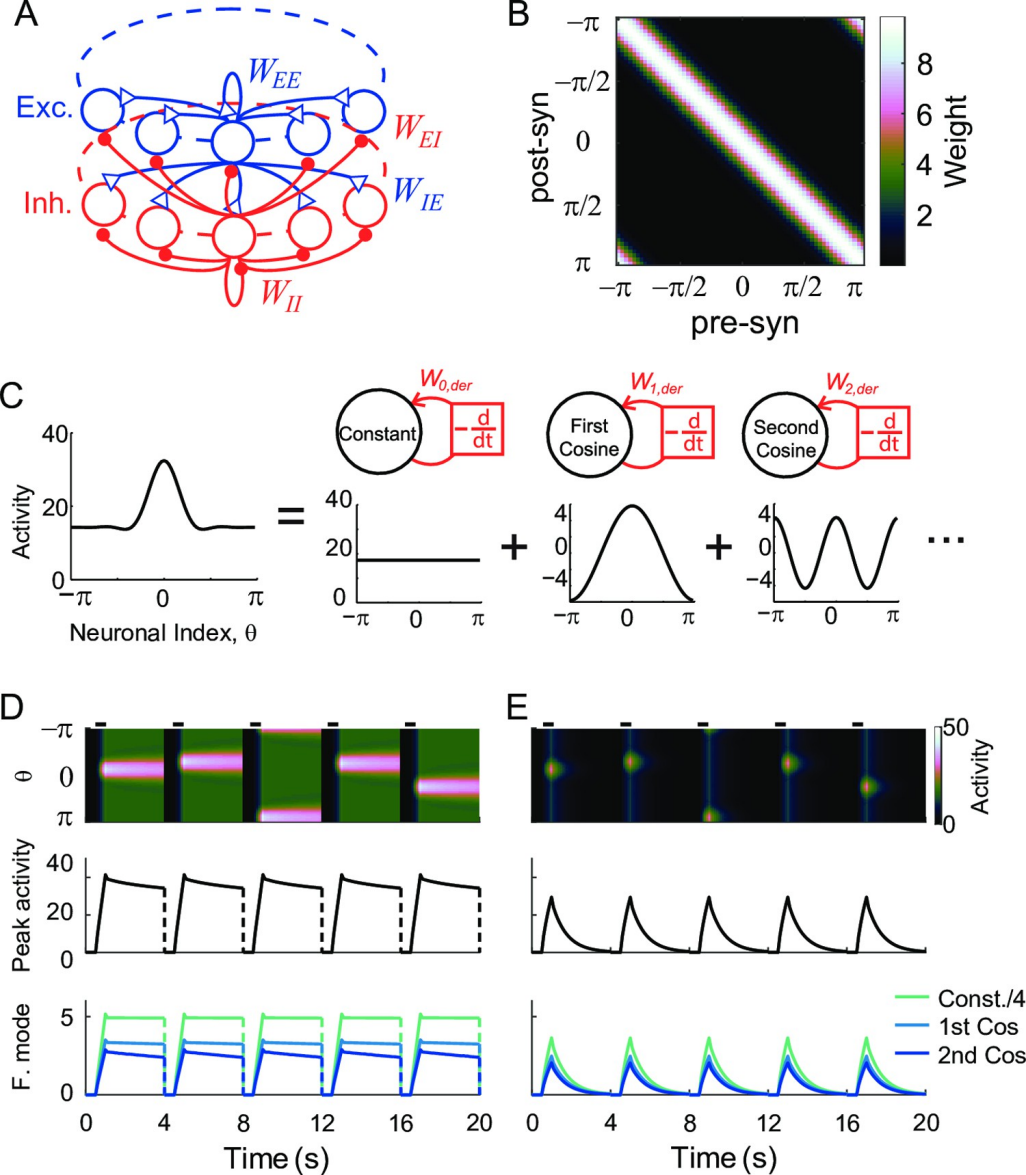
Location-coded persistent activity and its disruption under perturbation of tuning. A: Schematics of the spatial structure of network for location-coded memory. We considered that both excitatory and inhibitory neurons are organized in a columnar structure where each column consists of neurons with a similar preferred feature of the stimulus. Blue and red represent excitatory and inhibitory connections, respectively. B: Example connectivity matrix showing symmetry under translation. We considered the memory neurons encode the spatial information during the delay period, which lies on a circle, represented by θ ranging between -π and π. We assumed that before perturbation, the synaptic strengths depend only on the difference between feature preference of post and presynaptic neurons. C: Decomposition of spatially patterned activity into Fourier modes under translation-invariance. Figure adapted from [13]. D-E: Location-coded persistent activity under E-I balance (D) and its disruption under 10% global perturbation in the E-to-E connection (E). The upper panels show the activity of all neurons during five consecutive trials with each neuron labeled by its preferred feature. The middle panels show the activity of the neuron at the stimulus center and the lower panels show the activity of 3 Fourier modes with the constant mode shrunk by a factor of 1/4 for better visualization.

Previous work showed that the principle for negative derivative feedback found for one homogenous population could be extended to a network with columnar structure. Such a structure is required to maintain a spatial pattern of persistent activity. Consistent with experimental observations [39–41], both excitatory and inhibitory neurons in each column have similar spatial selectivity. The connectivity strengths decrease as the preferred features over the columns get dissimilar (Fig 5A and 5B). Assuming translation invariance of connectivity strength such that it depends only on the distance between neurons’ preferred features, the network activity is symmetric under the translation of stimulus location. Note that as in [13], we consider a network encoding circular variables such as direction with periodic boundary conditions, so translation-invariance is equivalent to rotation-invariance on a ring.

Under translation-invariant connectivity and activity patterns, dynamics can be analyzed through Fourier analysis, where the spatial pattern of the population activity is decomposed into a sum of cosine modes (Fig 5C; [13]). Assuming linear dynamics of neurons, recurrent synaptic inputs can be broken into the product of synaptic strengths and activity in each cosine mode. Then the dynamics of each mode are analogous to the dynamics of one homogeneous population (Methods). Although not considered here, even in the presence of nonlinear input-output transfer function, the dynamics under strongly balanced excitation and inhibition are similar to linear dynamics [12,13,34]. Thus, the condition for negative derivative feedback in each Fourier mode is similar to the rate-coded network—slower recurrent excitation with the same condition on the synaptic time constants as in the homogeneous case, and balanced recurrent excitation and inhibition of that mode represented in terms of the Fourier coefficients of the synaptic strengths. With excitation and inhibition balanced in each Fourier mode, the spatial pattern of that Fourier mode can be stabilized during the delay period [13].

With similar balanced tuning conditions for the location-coded persistent memory, the perturbation to the synaptic connections leads to a similar disruption in the activity as in the rate-coded network (Fig 5D and 5E). We first considered the multiplicative scaling down of all E-to-E connections, called a global perturbation (Fig 5E). This leads to imbalanced excitation and inhibition and decay of activity in all Fourier modes. Note that the translation-invariant property is maintained under the global perturbation of the connectivity. Thus, the activity pattern is still symmetric for different stimulus locations despite its rapid decay to the baseline compared to the unperturbed case (Fig 5E).

### Effects of differential plasticity under global perturbation

Next, we examined whether differential plasticity can recover the balance tuning condition for a spatially structured network. We assumed that the stimulus location is uniformly distributed and changes randomly across different trials. As in the homogeneous population, plasticity rules were applied during the delay period in each trial.

**Fig 6:**
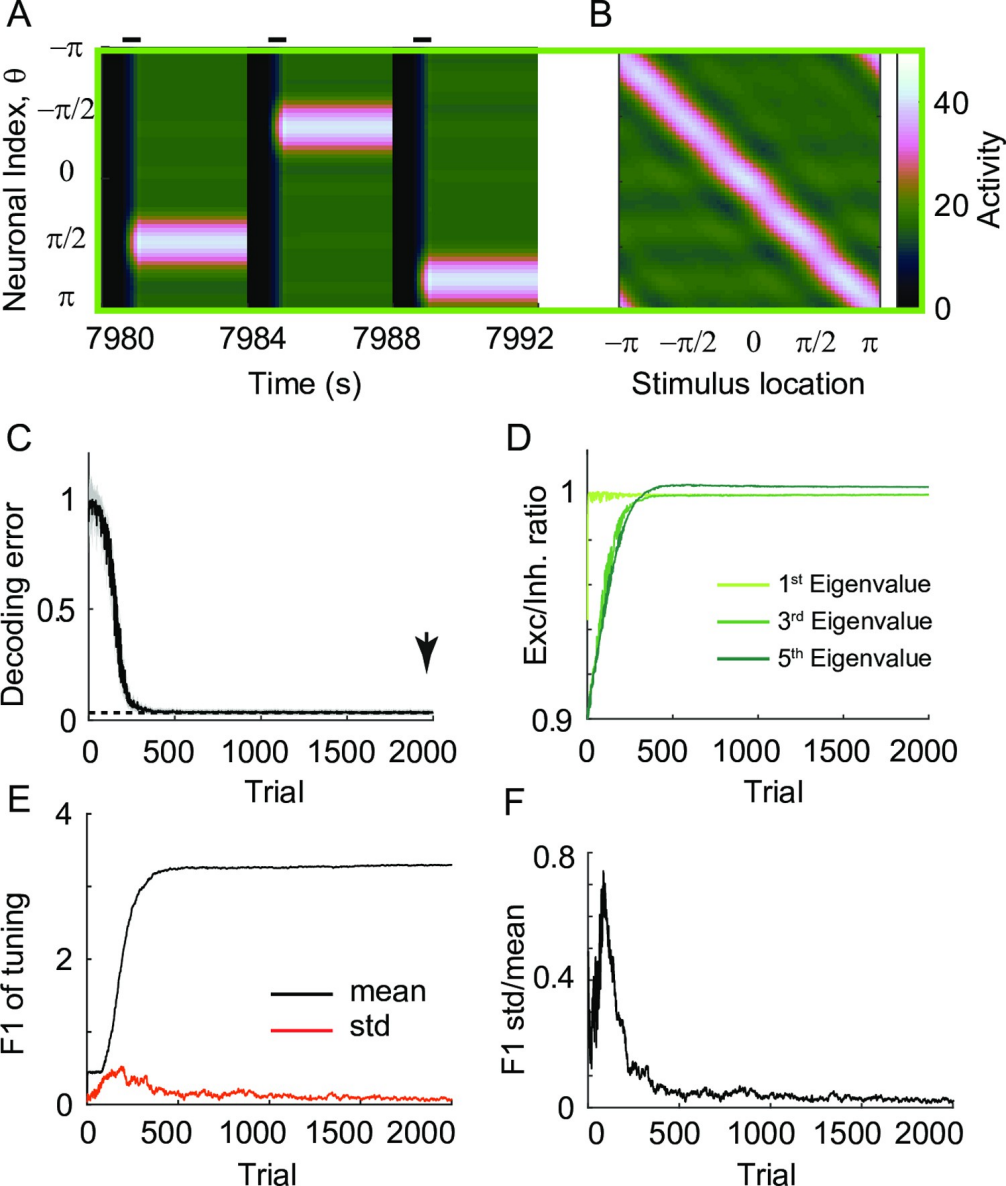
The effect of differential plasticity under small global perturbation. A: Recovery of location-coded memory under differential plasticity with learning rate α_*d*_ = 10^−3^ and 10% global perturbation in the E-to-E connections. B: Activity pattern at the end of the delay period after the recovery. With the connectivity frozen at trial 2000 (arrow in C), the spatial pattern of activity at the end of the delay period was shown for different stimulus locations. C: Decrease of decoding error with learning. An individual trial refers to one memory task with a specific stimulus location. For each trial, we took the snapshot of activity at the end of the delay period as in B and quantified the mean of the decoding error using the population vector decoder (black curve; Methods) and the standard error of the mean (grey shaded area). Dashed line indicates decoding error before perturbation. D: Recovery of E-I balance for different Fourier modes. The eigenvector decomposition reveals the effective time constant of decay and recovery of E-I balance in different Fourier modes (S2 Fig; Methods). E: Mean (black) and standard deviation (red) of spatial selectivity across neurons quantified by the first Fourier component of each neuron’s tuning curve at the end of the delay period. F: Normalized standard deviation of spatial selectivity in (E), where its decrease with learning indicates recovery of translation-invariance.

For a small global perturbation, the differential rule was shown to recover persistent activity in forms of spatial patterns like the ones before perturbation (Fig 6). Unimodal activity peaked at the stimulus location can be maintained at any location after the differential plasticity rule recovers the balance of excitation and inhibition (Fig 6A and 6B). We quantified the ability to maintain location-coded persistent memory using the decoding error of spatial information at the end of the delay period (Methods). Initially, after global perturbation, the decoding error became around one, indicating loss of spatial information. Over the course of learning with differential plasticity, it becomes close to the decoding error before the perturbation (Fig 6C). In line with this, the time constant of decay of different Fourier modes was shown to prolong (S2 Fig). In the eigenvector decomposition of the connectivity matrix, eigenvectors corresponding to the leading eigenvalues were found to be similar to Fourier modes towards the end of the simulation, which is a signature of preservation of translation-invariance (S2 Fig; [42]). The ratios of associated eigenvalues increase to one, albeit with different speeds, suggesting the recovery of the balance tuning condition in each mode (Fig 6D).

**Fig 7:**
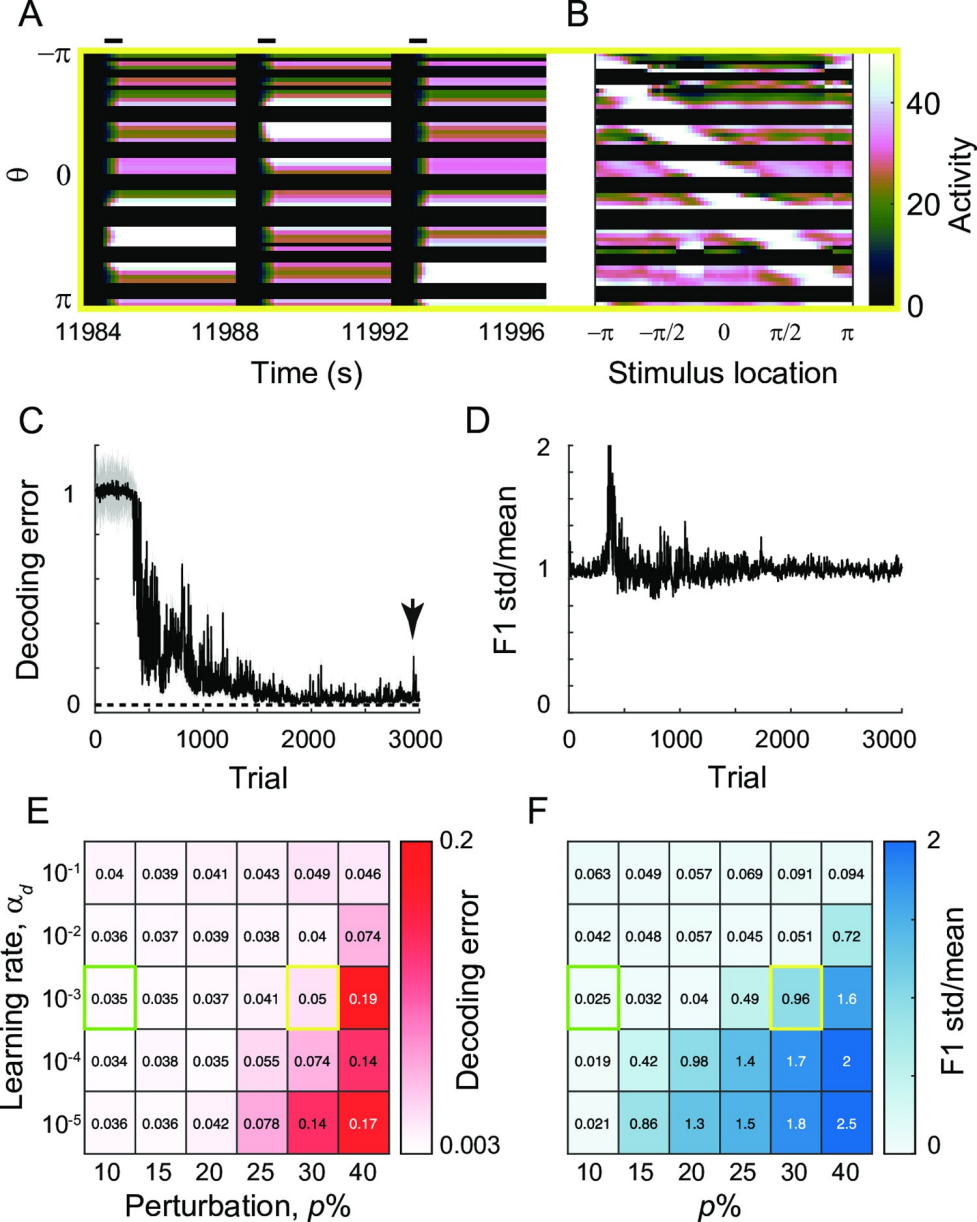
The effect of differential plasticity under various levels of global perturbation and learning rates. A-B: Activity pattern during three successive trials (A) and at the end of delay period for various stimulus locations (B) after reaching the steady state with α_*d*_ = 10^−3^ and 30% global perturbation (yellow box in E,F). C-D: Evolution of decoding error (C) and normalized deviation of spatial selectivity (D). E-F: Heatmap showing decoding error (E) and normalized deviation of spatial selectivity (F) after reaching steady state under different learning rates α_*d*_ and perturbation strengths *p*. The green box and yellow box correspond to the case showing recovery of translation-invariance (Fig 6) and the case with breaking-down of translation-invariance (Fig 7A–7D).

However, if the perturbation is large, then translation invariance breaks down, and Fourier analysis cannot be applied. For larger perturbation, the persistence of activity is recovered under differential plasticity, but the spatial pattern is fragmented by silent neurons (Fig 7A and 7B). In these silent neurons, inhibition from neighboring neurons exceeds total excitatory inputs during the stimulus period, and due to threshold nonlinearity, translation-invariance breaks down. With no activity during the delay period, the activity-dependent differential plasticity cannot potentiate incoming recurrent excitation, and the recovery of persistent activity is not uniform across different neurons.

To quantify this heterogeneity, we calculated the first Fourier component of the tuning curve of each neuron at the end of the delay period, representing its spatial selectivity, and obtained its mean and standard deviation across neurons (Fig 6E; Methods). Its mean increases with learning, indicating the increase of spatial selectivity with learning (Fig 6E). The ratio between the mean and standard deviation was used to quantify the translation-invariance, because with a translation-invariance state, spatial selectivity is uniform across neurons, and its standard deviation is relatively small compared to the mean, leading to their ratio close to zero. On the other hand, this quantity is of order one if the translation-invariance breaks down [31].

For a small perturbation, the variance of the first Fourier component of the tuning curve can transiently increase, reflecting an overall increase of activity level. However, the normalized variance decreases over successive trials with the translation-invariance maintained (Fig 6E and 6F). For larger perturbation, a fraction of neurons becomes silent and the normalized variance of spatial selectivity is not reduced to zero even after decoding error reaches its asymptote, indicating the breakdown of translation-invariance (Fig 7D). Note that even with a loss of translation-invariance, the decoding error can still be low (Fig 7C) in the case that only a few neurons are silent and the neighboring neurons partially compensate for them with enhanced rates. However, as more neurons get silent under larger perturbation, the network eventually loses the ability to encode and maintain spatial information (Figs 7E, 7F and S3).

We also explored how the decoding error and translation-invariance improve under differential plasticity as varying the learning speed α_d_ (Fig 7E and 7F). Numerically, it was found that decreasing α_*d*_ provides a similar effect to increasing perturbation strength *p*. Either larger perturbation or slower learning rule tends to create more silent neurons and degrade both decoding performance and translation-invariance (S3 Fig). Note that such an inverse relationship between α_*d*_ and *p* is consistent with one observed in a homogeneous population (Fig 2B and 2E). Also, the effect of changing other network parameters, overall feedback strength *W*_*inh*_ or input strengths *c*, can be inferred from the effect of α_*d*_ based on the relationship found in the homogeneous case.

### Effects of homeostatic plasticity under global perturbation

**Fig 8:**
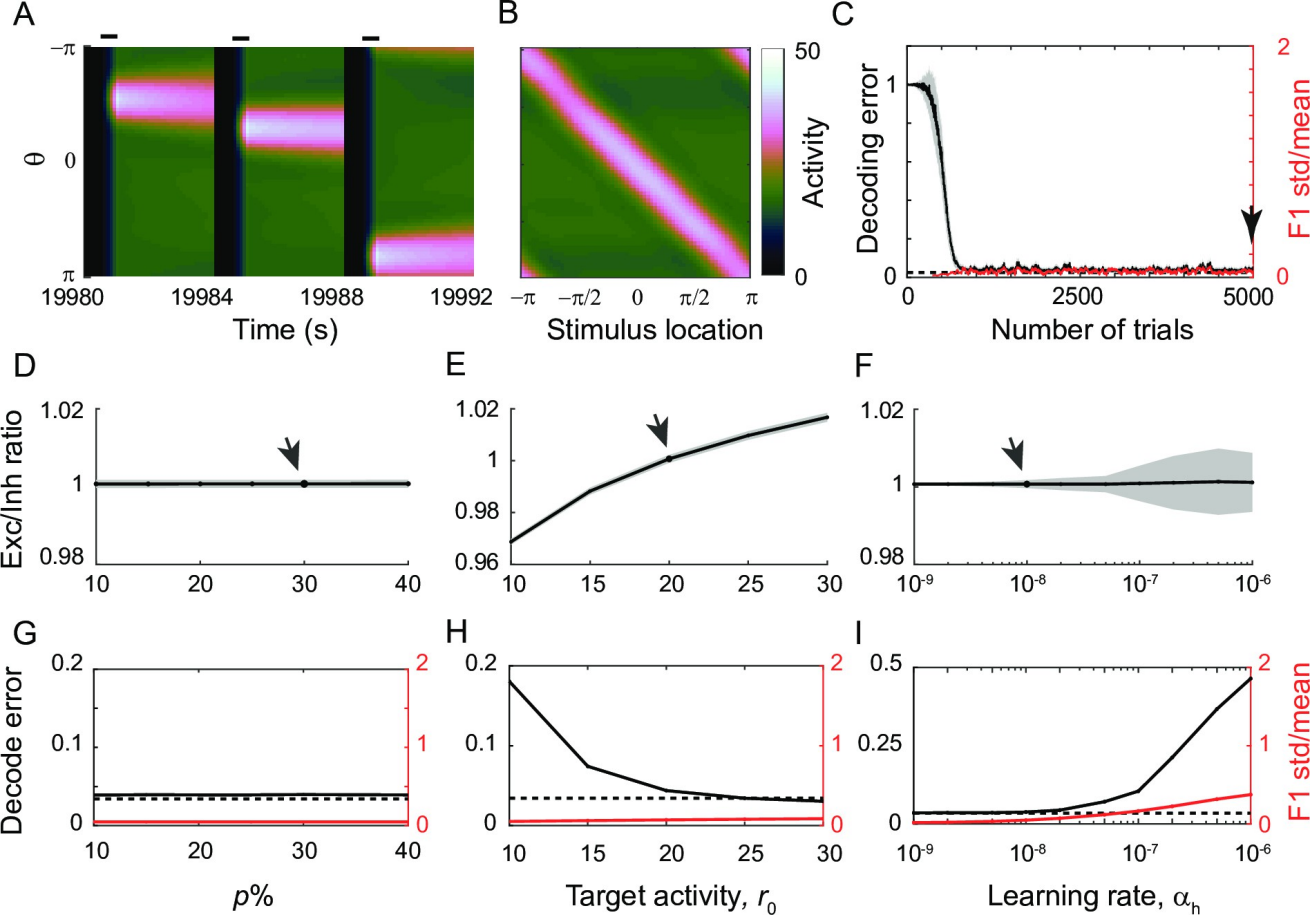
The effect of homeostatic plasticity under global perturbation. A: Recovery of location-coded memory under homeostatic plasticity with target rate *r*_*0*_ = 20, learning rate α_*h*_ = 10^−8^ and perturbation strength *p* = 30% in the E-to-E connections (arrow in D-F). B: Activity pattern at the end of the delay period after the recovery (arrow in C). C: Decrease of decoding error (black) and preservation of translation-invariance (red) with learning. D-F: Dependence of postsynaptic E-I ratio on target firing perturbation strength *p* (D), rate *r*_*0*_ (E), and learning rate α_*h*_ (F). Note that, unlike Fig 6D, the E-I ratio is not defined by eigenvalue or in the Fourier domain. As homeostatic plasticity modifies all incoming synapses of a neuron with a common factor, we quantified the E-I ratio compared to that before perturbation for each neuron. The mean is shown in black, and the standard deviation across neurons is shown in grey shaded area. G-I: Decoding error and normalized deviation of spatial selectivity for various *p* (G), *r*_*0*_, (H), α_*h*_ (I).

While the above results show that the maintenance of translation-invariance is not guaranteed under differential plasticity, homeostatic plasticity has been suggested to restore translation-invariance after it has been perturbed under heterogeneity of cellular excitability or synaptic inputs, or by other types of synaptic plasticity such as Hebbian learning [30,31]. Indeed, the application of a homeostatic learning rule to the negative derivative feedback network recovers persistent unimodal activity at different locations as well as translation-invariance (Fig 8A, 8B, and 8C). Unlike differential plasticity, such a recovery was less affected by changes of perturbation strengths (Fig 8D and 8G).

However, as in the homogeneous population, the steady state of homeostatic learning is sensitive to target activity *r*_*0*_ (Fig 8E and 8H). If *r*_*0*_ is too high (low) compared to the input strengths, the excitatory synapses are more potentiated (depressed) than the balance tuning condition (Fig 8E). Note that although such imperfect balance between excitation and inhibition results in an increase (decrease) of activity amplitude, the spatial information encoded in the peak of spatial pattern can be maintained unless *r*_0_ is too low (Fig 8H). For higher *r*_0_, the decoding error even gets reduced because of the enhanced firing rate.

Another learning parameter that affects the steady state of homeostatic learning is learning speed α_*h*_. Numerical simulation showed that the balanced tuning condition, decoding performance, and translation-invariance recover for a wide range of α_*h*_ (Fig 8F and 8I; Note the log-scale of x-axis). However, as in the homogeneous population, too fast α_*h*_ can lead to larger variability near the balanced state (Fig 8F). As a result, the E-I ratio oscillates and varies across the cells, causing drift of the activity bump and estimated location along the ring within a single trial (S4 Fig). This drift increases decoding error and breaks down translation-invariance (Fig 8I).

### Effects of plasticity under local perturbation

**Fig 9:**
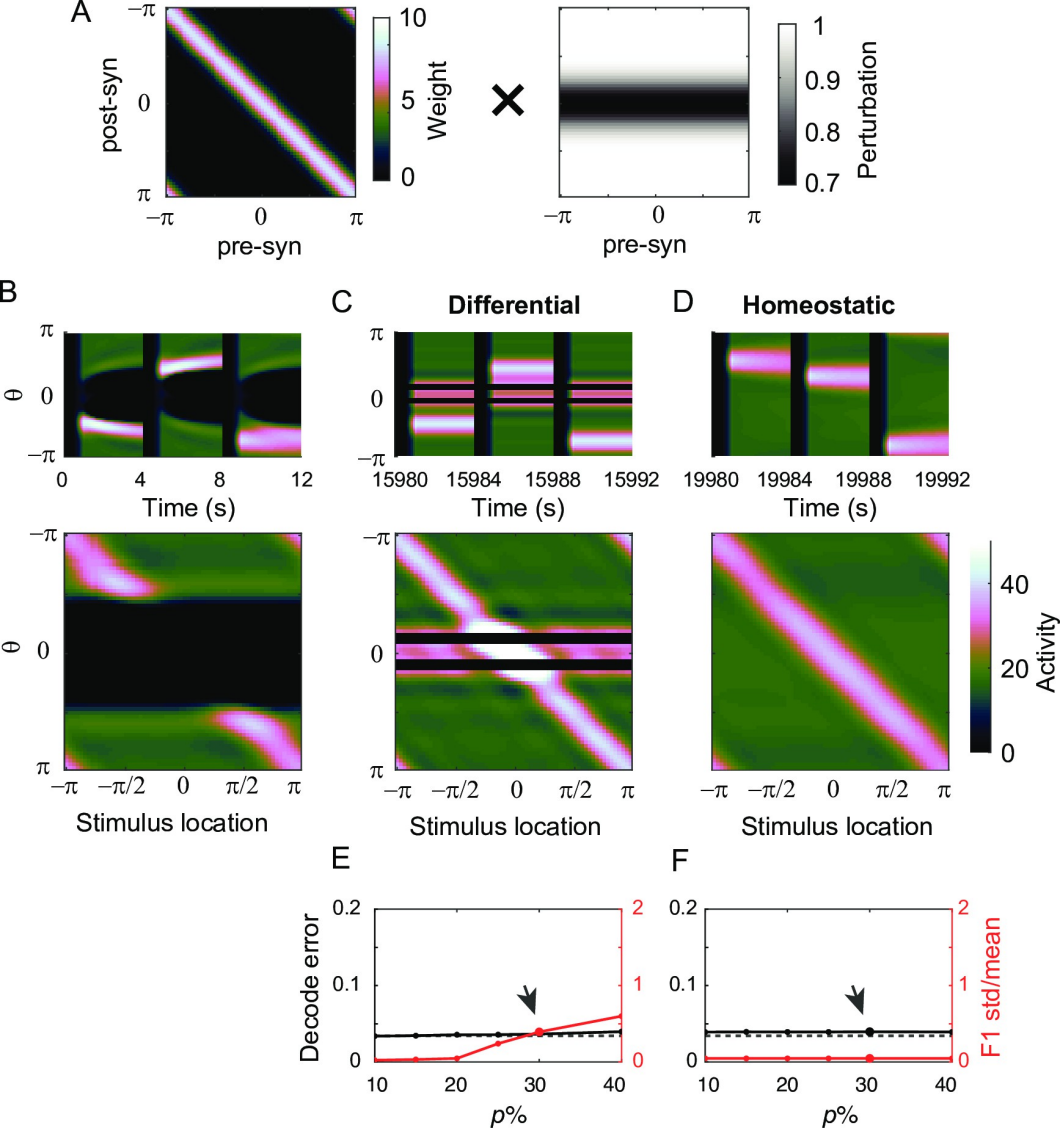
The effect of differential and homeostatic plasticity under postsynaptic perturbations. A: Schematics of postsynaptic perturbations where the rows of the connectivity matrix are multiplied by different scaling factors. Perturbation is centered at θ = 0 and bell-shaped. B: Activity pattern under 30% postsynaptic perturbations before any plasticity. C-D: Activity pattern shaped by the differential (C) and homeostatic (D) plasticity. The learning parameters used here are α_*d*_ = 10^−3^, α_*h*_ = 10^−8^, and *r*_*0*_ = 20. E-F: Decoding errors (black) and normalized deviation of spatial selectivity (red) for different perturbation strengths after applying differential (E) and homeostatic (F) plasticity. Perturbation strength marked by arrow is shown in C-D.

**Fig 10:**
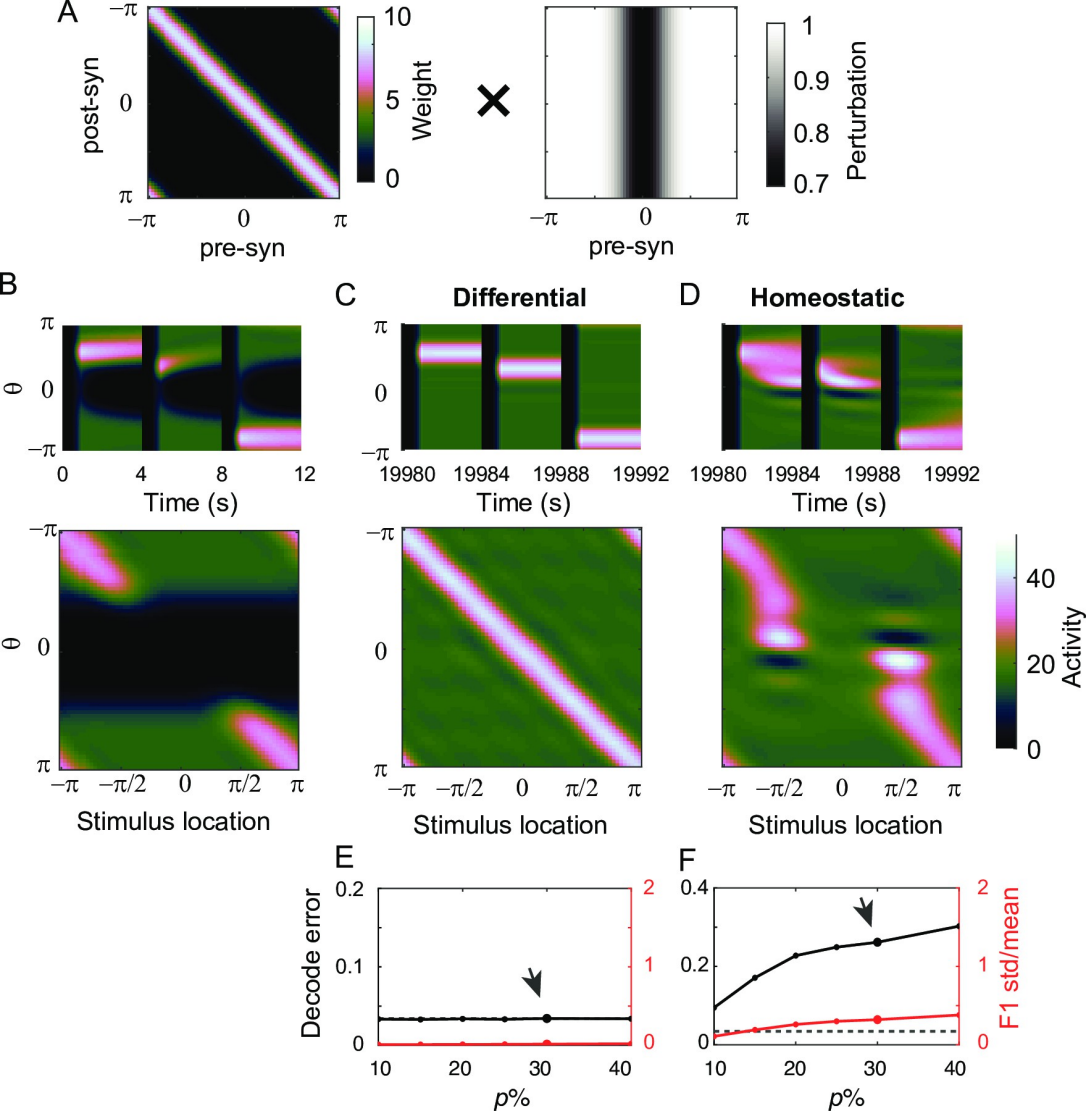
The effect of differential and homeostatic plasticity under presynaptic perturbations. A: Schematics of presynaptic perturbations where the columns of the connectivity matrix are multiplied by different scaling factors. B-F: Same as in Fig 9B–9F but under 30% presynaptic perturbation (B-D) and the same learning parameters.

We further investigated the effect of differential and homeostatic plasticity, where the balance of excitation and inhibition is locally perturbed. We considered two different types of local perturbations–first, postsynaptic perturbations, where synaptic strengths projected onto a particular group of neurons were perturbed (Fig 9). For instance, this can be incurred by perturbation in NMDA receptors, which is considered to be prominent in the E-to-E connections [43]. Mathematically, it is analogous to a row-wise perturbation in the E-E connectivity matrix (Fig 9A). Another type of perturbation is the presynaptic one, where outgoing synaptic strengths are perturbed (Fig 10). This perturbation can be caused by reducing transmitter release and is analogous to column-wise perturbation in the connectivity matrix (Fig 10A).

Note that we considered only network-wise perturbations because in the rate model considered in this study, an individual unit corresponds to a population of neurons with shared selectivity and the effect of perturbations on individual neuronal levels cannot be explored. For instance, random fluctuation of individual synapses has been observed on a daily basis [44]. If such fluctuation is uncorrelated across neurons, persistent memory may not be affected because the tuning condition in the rate model only constrains the population-averaged synaptic strengths while allowing heterogeneity in individual neurons [12]. Thus, we focused on network-wise perturbations, particularly a smooth bell-shaped perturbation of pre- or post-synaptic strengths, assuming that the neurons with similar preferred spatial selectivity are clustered, and the effect of local perturbation dissipates across the clusters [45].

We first examined the effect of plasticity in postsynaptic perturbations. In negative derivative feedback models, the postsynaptic perturbation disrupts local E-I balance, leading to quick decay of activity in the vicinity of the perturbed site (Fig 9B). Under a small perturbation, both differential and homeostatic plasticity can recover E-I balance and the ability to maintain persistent activity at the perturbed site (Fig 9E and 9F). However, when the perturbation becomes larger, differential and homeostatic plasticity show different recovery patterns as for the global perturbation (Fig 9C and 9D). For larger perturbation, differential plasticity persistently silences more neurons, which breaks down translation-invariance (Fig 9C and 9E). Note that as under global perturbations, slow learning speed or larger postsynaptic perturbation can also disrupt the decoding performance, while faster differential plasticity can mitigate the disruption caused by larger postsynaptic perturbation (S5 Fig). In contrast, homeostatic plasticity efficiently recovers translation-invariance for a wide range of perturbation strengths (Fig 9D and 9F). This is because homeostatic plasticity multiplicatively scales up the overall incoming synaptic strengths onto particular neurons when their activity is lower than *r*_0_ even if they decay to silence. This multiplicative scaling up exactly counteracts post-synaptic perturbation.

Next, we considered the effect of plasticity under presynaptic perturbations, which showed better performance of differential plasticity than homeostatic plasticity (Fig 10). As in the postsynaptic perturbations, presynaptic perturbation causes activity at the perturbed site to decay because perturbation in outgoing synapses mostly affects the incoming synapses of neurons with similar spatial selectivity (Fig 10B). Differential plasticity can recover persistent activity and translation-invariance for a broad range of presynaptic perturbation (Figs 10C, 10E and S6). On the other hand, homeostatic plasticity cannot stabilize persistent activity for relatively large presynaptic perturbation, and the activity pattern is distorted near the perturbed site (Fig 10D). This is because presynaptic perturbation introduces an asymmetry in the synaptic strengths projecting onto neurons near the perturbed sites, which cannot be recovered through homeostatic plasticity that regulates the overall scaling of incoming synapses. Thus, although the average postsynaptic activity is recovered through increased excitability, the bump activity drifts towards instead of away from the perturbed site after learning, leading to a high decoding error and breakdown of translation-invariance both (Fig 10F).

### Effect of combining differential and homeostatic plasticity

As differential plasticity and homeostatic plasticity are effective in recovering persistent activity and translation-invariance under the different types of perturbations, we examined whether the combination of these two plasticity rules can utilize the advantage of each plasticity. Following the previous models considering the combination of Hebbian and homeostatic plasticity [31], we considered a multiplicative combination of two rules where differential plasticity replaces Hebbian learning. The synaptic connection from neuron *j* to neuron *i* is expressed as a product of two variables, *W*_*ij*_ *= g*_*i*_*U*_*ij*_ with the dynamics of *g*_*i*_ and *U*_*ij*_ are given as

$$\begin{array}{c} \frac{dg_{i}}{dt}=-\alpha_{h}(r_{i}-r_{0})g_{i}\\ \frac{dU_{ij}}{dt}=-\alpha_{d}\frac{dr_{i}}{dt}r_{j}. \end{array}$$ (4)

In the above equations, *g*_*i*_ reflects the homeostatic scaling, and *U*_*ij*_ evolves according to differential plasticity, with the learning rates given as α_*h*_ and α_*d*_, respectively. Note that a multiplicative combination can be approximated by an additive combination and thus, can have similar effects (Methods).

**Fig 11:**
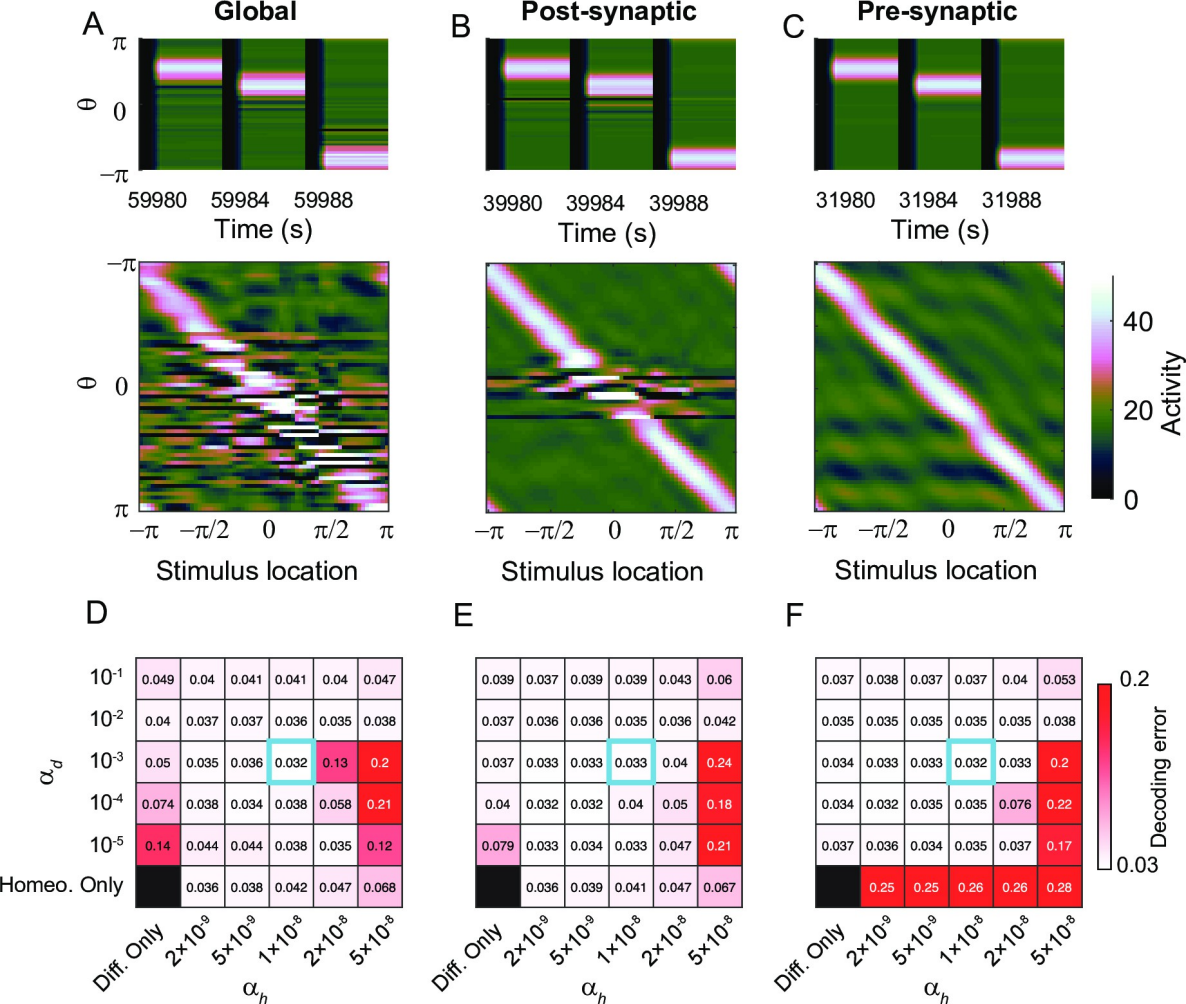
The effect of the combination of differential and homeostatic plasticity. A-C: Recovery of location-coded persistent activity under combined plasticity after 30% global (A), postsynaptic (B), and presynaptic perturbation (C) with the same learning parameters in Figs 9 and 10. The combined plasticity shows better performance compared to the recovery with differential plasticity alone under global perturbation (Fig 7A–7D), under local postsynaptic perturbation (Fig 9C) and the recovery with homeostatic plasticity alone under local presynaptic perturbation (Fig 10D). D-F: Heatmap of the final decoding error under various learning speeds. See S7–S9 Figs for normalized deviation of spatial selectivity and the activity pattern from which decoding errors and spatial selectivity variability were derived.

We first examined the effect of combined plasticity under global and postsynaptic perturbations. We considered large perturbations under which differential plasticity alone leads to the silence of activity (Figs 7A–7D and 9C). On the other hand, homeostatic plasticity prevents silent neurons by boosting lower-than-target activity. Thus combined plasticity could recover the network from larger global and postsynaptic perturbation (Fig 11A and 11B; left-most column vs. next three columns in Fig 11D and 11E).

Note that such a recovery is sensitive to α_h_ such that combined plasticity with large α_*h*_ performs worse than differential plasticity or homeostatic plasticity alone (right-most two columns in Fig 11D and 11E). This is because fast homeostatic plasticity leads to oscillation of synaptic weights near the steady state and the activity drift during the delay period (Fig 8F and 8I). Such activity drift can conflict with differential plasticity and lead to more disruption in spatial pattern of activity compared to differential plasticity or homeostatic plasticity alone (S7 and S8 Figs). Thus, slow homeostatic plasticity is required to enhance the decoding performance for a broad range of the learning speed of differential plasticity α_*d*_.

The superiority of the combined plasticity is similar for presynaptic perturbations (Fig 11C and 11F). Under large presynaptic perturbations, homeostatic plasticity alone could not restore memory performance, while differential plasticity was effective (Fig 10C and 10D). The combined one shows better memory performance compared to homeostatic plasticity alone (bottom row vs. the rest of rows in Fig 11F). Again, such improvement can be achieved unless homeostatic plasticity is too fast (right-most column in Figs 11F and S9).

## Discussion

In this work, we investigated the effects of local and unsupervised learning on the stabilization of persistent activity in two representative working memory models encoding analog values, namely, rate-coded and location-coded persistent memory. We examined the effects of differential plasticity and homeostatic plasticity by systematically varying the learning parameters and the magnitude and form of perturbations in synaptic connections. Consistent with the findings of previous works, differential plasticity alone was enough to stabilize a graded-level persistent activity in a homogeneous population [12,19]. On the other hand, homeostatic plasticity requires the tuning of learning parameters. For the maintenance of spatially structured persistent activity, differential plasticity could stabilize persistent activity, but its pattern can be irregular for different stimulus locations. Homeostatic plasticity shows a robust recovery of translation-invariance against particular types of synaptic perturbations, such as perturbations in incoming synapses onto the entire or local populations. However, homeostatic plasticity was not effective against perturbations in outgoing synapses from local populations. Instead, combining it with differential plasticity recovers the location-coded persistent activity for a broader range of perturbations.

Different parameter dependence of the two learning rules can also be used to distinguish them experimentally. First, varying perturbation strength provides a different prediction on recovery of persistent activity under the two learning rules. Experimentally, the magnitude of perturbation can depend on the proportion of perturbed neurons because neuronal activities and synaptic weights in the rate model are population-averaged. For instance, NMDA perturbation in a larger subpopulation results in larger perturbation *p* in the E-to-E connection. Note that although typical Hebbian-type synaptic plasticity depends on NMDA receptors [46], plasticity is intact in the unperturbed subpopulation with shared stimulus selectivity, and thus, can compensate for the perturbation as predicted from the rate model. Our study suggested that the emergence of silent neurons during recovery from large *p* indicates that differential plasticity may be dominant because the form of recovered persistent activity is less affected by *p* under homeostatic plasticity.

Another important parameter we explored is learning speed α. While direct manipulation of α might not be feasible, we showed that changing input strength *c* and overall inhibitory feedback strengths *W*_*inh*_ have a similar effect of changing α (Figs 2 and S1; Methods). Experimentally, *c* or *W*_*inh*_ can be varied through changes in gain/excitability of neurons or overall synaptic connections [47]. For instance, increasing the gain of excitatory neurons results in an increase of the effective strengths of incoming synapses onto excitatory neurons, leading to an increase of *W*_*exc*_, *W*_*inh*_ and *c*. We showed that the learning speed α, as well as the target rate of homeostatic plasticity, is expressed as the ratio of *c* and *W*_*inh*_, which remains constant (Eq 12). Thus, the recovery under homeostatic plasticity will not be affected. On the other hand, the different functional dependence of α on *c* and *W*_*inh*_ under differential plasticity leads to an effective decrease of α and slower recovery (Eq 10). Thus, the gain control of the excitatory population affects differential plasticity, but not homeostatic plasticity. Note that such parameter dependence of each plasticity was derived in negative derivative feedback models where the tuning condition is represented as the ratio of the feedback strengths, *W*_*exc*_ and *W*_*inh*_. With a proportional change of *W*_*exc*_ and *W*_*inh*_, perturbation strength *p* remains the same, and initial memory performance right after the perturbation would not be affected by the gain control of neurons. On the other hand, in positive feedback models where the tuning condition is represented as the difference of *W*_*exc*_ and *W*_*inh*_, changing the gain may lead to larger perturbation from the perfect tuning and more gross disruption of persistent activity right after the perturbation.

Stable memory formation under the mixture of different forms of synaptic plasticity has been proposed previously, mainly for discrete attractor networks [37,38,48,49]. In these studies, Hebbian synaptic plasticity has been suggested to form auto-associative memory guided by external inputs. To prevent instability caused by Hebbian learning, compensatory mechanisms, such as homeostasis or short-term plasticity, were required, which must act on a timescale similar to that of Hebbian learning ([50]; but see [49]). Our work also suggests synergistic interplay between different types of plasticity, differential and homeostatic plasticity, in particular for stabilizing location-coded persistent memory. However, we note that differential plasticity alone is stable. The role of homeostatic plasticity is to support translation-invariance in a ring-like architecture of recurrent connections [30,31]. Thus, the fast dynamics of homeostatic plasticity are not required, and excessively fast dynamics can be detrimental due to oscillatory instability. The interplay between anti-Hebbian learning and activity-dependent synaptic scaling has been proposed for rate-coded persistent memory [51], where the anti-Hebbian rule itself stabilizes the network activity and no fast homeostasis is required, as in our work.

In this work, we assumed the existence of synaptic plasticity only during the delay period. Continuous learning with homeostatic plasticity may require the adjustment of learning parameters because the long-term average firing rates of neurons must reflect activity during the entire session. On the other hand, differential plasticity might make the network “unlearn” if it operates the same way during the stimulus period as in the delay period because the activity rise during that time would be interpreted as positive drift by the plasticity. Thus, we constrained derivative-driven learning only during the delay period when the activity should be stabilized, as in [19]. One way to realize this is gating plasticity with the external input. For instance, Nygren et al. [20] proposed a network model for an oculomotor integrator that receives feedback from the “teacher” circuit. During the inter-saccadic interval, the teacher circuit provides low-pass filtered feedback so that its deviation from the instantaneous feedback provides a derivative-like signal that can guide self-supervisory learning as differential plasticity. On the other hand, the saccadic velocity input to the teacher circuit is tuned to make the self-supervisory signal zero, thus gate the plasticity during saccades. Alternatively, suppression of plasticity during the stimulus presentation could occur by filtering fast-changing activity [18]. We considered a similar possibility that there exists an upper bound of derivatives that can be sensed by the learning mechanism. With shortened duration and adjusted input strength so that the neural activity changes disproportionally faster in the stimulus period than in the delay period, the persistent activity could be stabilized even when the plasticity is always on ([12]; S10 and S11 Figs). How the derivative is sensed and filtered/saturated is beyond the scope of this paper and needs to be further investigated.

Constraining activity drifts of individual neurons might require stricter conditions than what is required to achieve stable coding of information during the memory period. While traditional experimental work identified memory neurons that showed persistence elevated firing with stimulus selectivity [45], the recent population-level analysis revealed the stable readout of information across various time points despite the diverse temporal dynamics of individual neurons [52,53]. Such dynamic activity in individual neurons may reflect activity in a downstream population that combines stimulus-encoding persistent activity and time-varying activity, possibly reflecting time information [54,55]. On the other hand, memory networks themselves can allow time-varying activity. For this attractor dynamics, the particular activity pattern or mode encodes persistent memory, while other modes allow temporal fluctuation [53,56]. For the latter, synaptic plasticity based on the global error signal has been suggested, which can be a self-supervised signal, such as a drift in the readout activity [21] or a difference from the target signal [57]. Note that the resulting form of synaptic plasticity is similar to differential plasticity, where the activity drift of individual neurons in differential plasticity is replaced with the global error signal. Homeostatic processes, such as intrinsic plasticity, inhibitory plasticity, and synaptic scaling, have also been proposed to elongate memory traces in the presence of dynamic activity [51,58]. In these works, the memory is maintained by a network with minimally structured connectivity, and the sensitivity to learning parameters has not been analyzed.

Overall, our work demonstrates how unsupervised learning can mediate fine-tuning conditions for working memory implemented by continuous attractors. It aligns with previous works emphasizing the role of unsupervised learning to generate a basis of activity patterns and dynamics underlying cognitive functions [59–61]. While we focused on unsupervised learning rules regularizing temporal patterns in the absence of input, they can be combined with other learning rules that can act under the guidance of external inputs and may make memory networks robust for a broader range of perturbations. Also, we considered perturbation and synaptic plasticity only in a specific connection, recurrent E-to-E connections, but the plasticity of other connections, such as inhibitory plasticity [62–64], has been suggested to tune network homeostasis and EI balance. Given the importance of balance and homeostasis in memory circuits, further investigation is needed to examine the effect of unsupervised plasticity on various synapses. Also, to understand how the learning parameters of these plasticity rules match with neural activity, a detailed investigation of the underlying biophysical mechanisms needs to be done, possibly in models involving multiple subcellular compartments.

## Methods

Here, we describe models of network and plasticity rules considered in our study, mathematical analysis, and parameters for the simulation. We first discuss a homogeneous population suggested for rate-coded persistent memory and then spatially structured networks for location-coded memory. For a homogeneous population, the models and simulation protocol were described in detail in the first three sections of the Result. Here we show mathematical analysis deriving a one-dimensional equation, parameter dependence, and parameters used in the simulation. For location-coded memory, we first describe network models and perturbation and plasticity models. Next, Fourier analysis for spatially structured networks, quantification of memory performance, and parameters are given. For numerical simulation, all codes are available at https://github.com/jtg374/NDF_ringNet_plasticity

### Simple rate model for a homogeneous population

In this section, we show the derivation of a one-dimensional differential equation in Eq 1 (see more biological structure and conditions in [12]). For this, we considered one homogeneous population receiving recurrent excitation and inhibition with different kinetics, described by three-dimensional differential equations

$$\begin{array}{c} \tau \frac{dr}{dt}=-r+W_{exc}s_{exc}-W_{inh}s_{inh}+I(t)\\ \tau_{exc}\frac{ds_{exc}}{dt}=-s_{exc}+r\\ \tau_{inh}\frac{ds_{inh}}{dt}=-s_{inh}+r, \end{array}$$ (5)

where three dynamic variables are firing rate *r*, recurrent excitatory currents *s*_*exc*,_ and recurrent inhibitory currents *s*_*inh*_. We assumed that *s*_*exc*_ and *s*_*inh*_ are low-pass filtered *r* with time constants τ_*exc*_ and τ_*inh*_, respectively.

Note that the feedback of the same strength but with time constants, *s*_*exc*_*−s*_*inh*_, can approximate the time-derivative of a signal, *dr/dt*, for low-frequency responses characteristic of persistent activity. To show this, we use the Laplace transform such that

$$L(s_{exc}-s_{inh})=\frac{R(u)}{\tau_{exc}u+1}-\frac{R(u)}{\tau_{inh}u+1}=-\frac{(\tau_{exc}-\tau_{inh})u}{\left(\tau_{exc}u+1)(\tau_{inh}u+1\right)}R(u),$$ (6)

where *R*(*u*) is the Laplace transform of *r*(*t*), and *u* is the complex-valued frequency. For low frequencies *u*, *L* (*s*_*exc*_*—s*_*inh*_) ≈ - (*τ*_*exc*_*—τ*_*exc*_) *uR*(*u*), that is, *s*_*exc*_*−s*_*inh*_ ≈ -(*τ*_*exc*_—*τ*_*inh*_)*dr/dt* as *L*(*dr/dt*) = *u*R(*u*).

With the difference between *s*_*exc*_ and *s*_*inh*_ approximating the time derivative of the activity and *s*_*exc*_ *≈ r* when *r* hardly changes, Eq 5 can be replaced with a one-dimensional differential equation, given as

$$\begin{array}{c} \tau \frac{dr}{dt}=-r+(W_{exc}-W_{inh})s_{exc}+W_{inh}(s_{exc}-s_{inh})+I(t)\\ \approx -r+(W_{\mathrm{exc}}-W_{inh})r-W_{inh}(\tau_{\mathrm{exc}}-\tau_{inh})\frac{dr}{dt}+I(t). \end{array}$$ (7)

With *W*_*exc*_*—W*_*inh*_ and *W*_*inh*_(τ_*exc*_—τ_*inh*_) denoted by *w*_*net*_ and *w*_*der*_, Eq 7 is the same as Eq 1. Such a one-dimensional approximation allows analytic investigation on the effects of differential plasticity and homeostatic plasticity in Eq 2 and Eq 3.

### Parameter dependence in a homogeneous population

Next, we examine the parameter dependence of recovery under differential plasticity and homeostatic plasticity after perturbations in connectivity strengths. For analytical tractability, we assumed *W*_*inh*_ is large such that 1/*W*_*inh*_ ~ 0, and we extracted the scale factor *c* from the input to investigate the effect of overall input strengths. Then Eq 7 becomes

$$(\tau_{exc}-\tau_{inh})\frac{dr}{dt}=\frac{\left(W_{exc}-W_{inh}\right)}{W_{inh}}r+\frac{c}{W_{inh}}\hat{I}(t).$$ (8)

In Eq 8, when *W*_*exc*_ and *r* are normalized with *W*_*inh*_ and *c*/*W*_*inh*_, denoted as *w = W*_*exc*_*/W*_*inh*_ and *r*_*n*_ *= r/(c/W*_*inh*_*)*, the dynamics with the differential plasticity in Eq 2 becomes

$$\begin{array}{c} \left(\tau_{exc}-\tau_{inh})\frac{dr_{n}}{dt}=(w-1)r_{n}+\hat{I}(t\right)\\ \frac{dw}{dt}=-\frac{\alpha c^{2}}{W_{inh}^{3}}\frac{dr_{n}}{dt}r_{n}. \end{array}$$ (9)

Thus, increasing *W*_*inh*_ has the same effect as decreasing α to the third power, and increasing *c* has the same effect as increasing α to the second power (Fig 2C and 2D).

Furthermore, the relationship between α and *p* can be revealed by integrating the second line in Eq 9 until the system reaches the steady state in a single trial as

$$\begin{array}{c} \int_{0}^{\infty }\frac{dw}{dt}dt=-\frac{\alpha c^{2}}{W_{inh}^{3}}\int_{0}^{\infty }\frac{1}{2}\frac{dr_{n}^{2}}{dt}dt\\ \to w(\infty )-w(0)=-\frac{\alpha c^{2}}{2W_{inh}^{3}}(r_{n}^{2}(\infty )-r_{n}^{2}(0)). \end{array}$$ (10)

If we assume that the final state of *w* is one corresponding to the balanced state and the initial perturbation is *p*, that is, *w*(0) = 1-*p*, then the left-hand side becomes *p*. Thus, the final state of *r*_*n*_ can be represented as

$$r_{n}^{2}(\infty )=r_{n}^{2}(0)-\frac{2W_{inh}^{3}p}{\alpha c^{2}}.$$ (11)

As the second term on the right-hand side only contains the ratio of α and *p*, increasing *p* results in the same final *r*_*n*_ as decreasing α with the same initial *r*_*n*_.

With the normalization of *W*_*exc*_ and *r* with *W*_*inh*_ and *c*/*W*_*inh*_, the dynamics with homeostatic plasticity in Eq 3 can be simplified as

$$\begin{array}{c} \left(\tau_{exc}-\tau_{inh})\frac{dr_{n}}{dt}=(w-1)r_{n}+\hat{I}(t\right)\\ \frac{dw}{dt}=-\frac{\alpha c}{W_{inh}}w(r_{n}-\frac{W_{inh}}{c}r_{0}). \end{array}$$ (12)

The recovery to the balanced state is affected by *r*_*0*_ but not by the learning speed α (Fig 4A and 4B). Note that in Eq 12, increasing *c* or decreasing *W*_*inh*_ is equivalent to increasing α while decreasing *r*_*0*_ together (S1A, S1B and S1F Fig).

Next, we explore how the stability near the steady state is affected by changing the learning speed α. In Eq 12, we consider *τ*_*exc*_*—τ*_*exc*_ *= 1* for simplicity and denote α*c/W*_*inh*_ and *W*_*inh*_*r*_*0*_*/c* as α’ and *r*_*0*_’, where α’ increases as α increases. Then the steady state of the dynamics given in Eq 12 is achieved when *r*_*n*_ = *r*_*0*_’ and *w* = 1 during the delay period with $\hat{I}(t)=0$. Then the eigenvalues of the Jacobian matrix at the steady state are $\pm i\sqrt{\alpha^{\prime }r_{0}^{\prime }}$. The imaginary part of eigenvalues reflects the frequency of oscillations. Thus, as α’ gets larger, the frequency increases, and the oscillation becomes prominent within each trial.

### Parameters for a homogeneous population

In Eqs 1–3, we set τ and τ_*exc*_*—*τ_*inh*_ to be unit time constant 1, and the durations of stimulus presentation, delay and inter-trial interval are 50-, 300-, and 50-time units, such that the total duration of one trial is 400-time units. Initial *W*_*exc*_, *W*_*inh*_ *and w*_*der*_ are set to be 500. *I(t)* is a step function with its strength randomly distributed as 0 and 1000 so that the mean input strength is 500 (Figs 1C–1F, 3 and 4), and *I(t)* for three representative traces of *r(t)* was 250, 500 and 1000 (Fig 1A). For the differential plasticity, the learning speed α is 0.01. For homeostatic plasticity, α is 4×10^−8^ in Figs 3, 4B and 4B and 4C, and 2×10^−6^ in Fig 4D. *r*_*0*_ is 50 in Figs 3 and 4D, 25 in Fig 4B, and 75 in Fig 4C.

### Spatially structured network model for location-coded persistent activity

Following [13], we considered a network organized in a columnar architecture for spatial working memory with the equations describing the dynamics given as

$$\begin{array}{c} \tau_{E}\frac{d}{dt}r_{E}(\theta )=-r_{E}(\theta )+q(\int_{-\pi }^{\pi }W_{EE}(\theta ,\theta^{\prime })s_{EE}(\theta^{\prime })d\theta^{\prime }-\int_{-\pi }^{\pi }W_{EI}(\theta ,\theta^{\prime })s_{EI}(\theta^{\prime })d\theta^{\prime }+I_{sp}(\theta ,\theta_{0})I_{temp}(t))\\ \tau_{I}\frac{d}{dt}r_{I}(\theta )=-r_{I}(\theta )+q(\int_{-\pi }^{\pi }W_{IE}(\theta ,\theta^{\prime })s_{IE}(\theta^{\prime })d\theta^{\prime }-\int_{-\pi }^{\pi }W_{II}(\theta ,\theta^{\prime })s_{II}(\theta^{\prime })d\theta^{\prime }), \end{array}$$ (13)

where subscripts *E* and *I* represent excitatory and inhibitory populations, respectively. The activity and the connectivity were indexed by their preferred spatial feature, θ, ranging between [-π,π). τ_E_ and τ_I_ are the time constants and *q(·)* is the input-output transfer function, which is the rectified linear function given as *q(x) = x* for *x > 0* and otherwise, 0. For numerical simulation, we considered *N* neurons for memory circuits with discretization of the spatial feature θ and approximation of integral in Eq 13 with summation over the number of neurons.

As in the homogeneous case, *s*_*ij*_ (*i*, *j* = E or I) represents the synaptic variables whose dynamics is given as

$$\tau_{ij}s_{ij}(\theta )=-s_{ij}(\theta )+r_{j}(\theta ).$$ (14)

Importantly, the excitatory-to-excitatory (E-to-E) time constant needs to be much larger than those of other synapses to make derivative feedback happen [12]. Detailed parameters used in the simulation will be given in Table 1.

**Table 1:** Parameters for spatially structured network.

Parameter	Description	Value
*N*	Number of populations in each E or I group	64
*τ*_*E*_	Time constant of excitatory neurons	20
*τ*_*I*_	Time constant of inhibitory neurons	10
*τ*_*EE*_	Time constant of E-to-E synapses	100
*τ*_*EI*_	Time constant of I-to-E synapses	10
*τ*_*IE*_	Time constant of E-to-I synapses	25
*τ*_*II*_	Time constant of I-to-I synapses	10
*τ*_*o*_	Time constant of external stimulus	100
*J*_*EE*_	Amplitude of E-to-E synaptic weight	100
*J*_*EI*_	Amplitude of I-to-E synaptic weight	100
*J*_*IE*_	Amplitude of E-to-I synaptic weight	200
*J*_*II*_	Amplitude of I-to-I synaptic weight	200
*J*_*o*_	Amplitude of external stimulus	270
*σ*_*EE*_, *σ*_*IE*_	Width of excitatory synaptic connections	0.2*π*
*σ*_*EI*_, *σ*_*II*_	Width of inhibitory synaptic connections	0.1*π*
*σ*_*o*_	Width of stimulus	0.25*π*
*h*_0_	Baseline of stimulus	200
*p*	1—perturbation strength	10%-40%
*α*_*d*_	Learning rate of differential rule	1e-5-0.1
*α*_*h*_	Learning rate of homeostatic rule	1e-9-1e-6
*r*_0_	Target firing rate of homeostatic rule	10–30
*t*_*stim*_	Stimulation duration	500
*t*_*total*_	Stimulation plus delay period	3500

W_ij_ (*i*, *j* = E or I) is the synaptic weight kernel, and before perturbation, it was taken to be translation-invariant and Gaussian-shaped as

$$W_{ij}(\theta ,\theta^{\prime })=J_{ij}\exp(-(d(\theta -\theta \prime ){)}^{2}/\sigma_{ij}^{2}),$$ (15)

where *d*(θ-θ’) = mod(|θ-θ’|,π) is the wrapped distance between θ and θ’. In practice we generate the center row of the weight matrices ${\overset{↔}{W}}_{ij}$ (with θ’ = 0 and θ ranging from -π to π-Δθ) and circularly shift it in other rows (see Fig 5).

*I*_sp_ (θ,θ_0_) and *I*_*temp*_(*t*) represent the spatial and temporal profiles of external stimulus where θ_0_ is the center of the stimulus location. *I*_sp_ (θ,θ_0_) is also a translation-invariant function that only depend on d(θ-θ_0_)

$$I_{sp}(\theta ,\theta_{0})=J_{o}\exp(-{\left(\frac{d(\theta -\theta_{0})}{\sigma_{o}}\right)}^{2})+h_{0}.$$ (16)

*I*_*temp*_(*t*) is a pulse function smoothed by a low-pass filter with time constant τ_o_ as in [12]:

$$I_{temp}(t)=\{\begin{array}{c} 1-\exp(-t/\tau_{o}),\;\mathrm{if}\;t<t_{stim}\\ I_{temp}(t_{stim})\exp(-(t-t_{stim})/\tau_{o}),\;\mathrm{if}\;t_{stim}\leq t<t_{total} \end{array}$$ (17)

where time within [0, *t*_*stim*_) refers to the stimulation period.

### Perturbation and plasticity model

We considered three types of perturbations in the E-to-E connections. For the global perturbation, ${\overset{↔}{W}}_{ij}$ was set to be

$${\overset{↔}{W}}_{EE,\mathrm{perturbed}}(\theta ,\theta^{\prime })=p_{\mathrm{uniform}}{\overset{↔}{W}}_{EE,0}(\theta ,\theta^{\prime }).$$ (18)

Postsynaptic perturbation corresponds to a row-wise change as

$${\overset{↔}{W}}_{EE,\mathrm{perturbed}}(\theta ,\theta^{\prime })=p_{\mathrm{post}‐\mathrm{syn}}(\theta ){\overset{↔}{W}}_{EE,0}(\theta ,\theta^{\prime }),$$ (19)

and presynaptic perturbation corresponds to a column-wise change as

$${\overset{↔}{W}}_{EE,\mathrm{perturbed}}(\theta ,\theta^{\prime })=p_{pre‐syn}(\theta^{\prime }){\overset{↔}{W}}_{EE,0}(\theta ,\theta^{\prime }),$$ (20)

where *p*(θ) is a smooth function of θ, given as a Gaussian function

$$p(\theta )=1-p\;\exp(-{\left(\theta /\sigma_{p}\right)}^{2}).$$ (21)

To recover the persistent activity, we considered two types of plasticity: differential plasticity,

$$\frac{dW_{ij}}{dt}=-\alpha_{d}\frac{dr_{i}}{dt}r_{j},$$ (22)

and homeostatic plasticity,

$$\frac{dW_{ij}}{dt}=-\alpha_{h}W_{ij}(r_{i}-r_{0}),$$ (23)

where α_*d*_ and α_*h*_ represent the learning rate of differential and homeostatic plasticity; *i* and *j* represent post- and presynaptic neuron index. Throughout the paper, except for S10 and S11 Figs, the plasticity is only applied in the delay period, and to minimize the effect of the residual stimulus, we also gated the plasticity with a factor 1-*I*_*temp*_(*t*), though it does not make much difference if we don’t add it.

In the combined one in Eq 4, *W*_*ij*_ in Eqs 22 and 23 are replaced by *U*_*ij*_ and *g*_*i*_, respectively. Note that the multiplicative combination can be approximated by additive combination because

$$\frac{dW_{ij}}{dt}=U_{ij}\frac{dg_{i}}{dt}+g_{i}\frac{dU_{ij}}{dt}$$

$$=-\alpha_{h}(r_{i}-r_{0})g_{i}U_{ij}-\alpha_{d}g_{i}\frac{dr_{i}}{dt}r_{j}$$

$$=-\alpha_{h}(r_{i}-r_{0})W_{ij}-\alpha_{d}g_{i}\frac{dr_{i}}{dt}r_{j},$$ (24)

where in the last equation, the first term is homeostatic plasticity, and the second term is differential plasticity with its speed α_*d*_*g*_*i*_. As *g*_*i*_ stays of order 1, the second term can be approximated by differential plasticity with constant speed.

### Fourier analysis and quantifying E-I balance through eigenvalue decomposition

When the connectivity is translation-invariant, i.e., *W*_*ij*_(θ,θ’) = *w*_*ij*_(d(θ-θ’)), the recurrent synaptic inputs in Eq 13 becomes the convolution between *w*_*ij*_(θ) and *s*_*ij*_(θ). In linear algebra, convolution can be represented by a product by a circulant matrix, whose normalized eigenvectors and eigenvalues are Fourier modes and corresponding Fourier coefficients [42]. Note that strongly balanced recurrent inputs make the network approximately linear [12,34]. Thus, in negative derivative feedback networks with strongly balanced excitation and inhibition, the dynamics can be analyzed through Fourier analysis.

Using the convolution theorem, the convolution in recurrent input can be expressed as a product of Fourier coefficients in the Fourier domain. In particular, if the dynamics are linear as *q*(*x*) = *x*, then Eq 13 becomes

$$\tau_{i}\frac{d{\hat{r}}_{i}(n)}{dt}=-{\hat{r}}_{i}(n)+{\hat{w}}_{\mathrm{iE}}(n){\hat{s}}_{\mathrm{iE}}(n)-{\hat{w}}_{\mathrm{iI}}(n){\hat{s}}_{\mathrm{iI}}(n),$$ (25)

where ${\hat{r}}_{i}(n),\;{\hat{w}}_{ij}(n)$ and ${\hat{s}}_{ij}(n)$ are the n-th Fourier coefficient of *r*_*i*_(θ), *w*_*ij*_(θ) and *s*_*ij*_(θ), respectively (*i*,*j* = *E* or *I*). Note the similarity between this equation and Eq 5.

In Figs 5D, 5E and S2A, we defined the *n*-th Fourier mode (*n* = 0 for constant mode) as

$$\hat{r}(n)=\frac{1}{2\pi }\int_{-\pi }^{\pi }r(\theta )\cos(n(\theta -\theta_{0}))d\theta ,$$ (26)

where θ_0_ is the stimulation center, and showed the example time course and estimated its timescale.

In Figs 6D and 8E, we quantify the recovery of EI balance by taking the eigenvalues of the weight matrices. When translation-invariance is preserved, the values of both E-to-E matrix and other weight matrices will approximately be the Fourier components of the matrices, and the tuning condition for the *n*-th Fourier modes becomes

$$\lambda_{EE}(n)\lambda_{II}(n)=\lambda_{EI}(n)\lambda_{IE}(n),$$ (27)

where λ_*ij*_(*n*) is the *n*-th eigenvalue of ${\overset{↔}{W}}_{ij}$. In Fig 6D, we did the eigenvector decomposition of the weight matrix ${\overset{↔}{W}}_{EE}$ and found the eigenvectors resemble Fourier modes and calculated the E-I balance ratio in each mode from the corresponding eigenvalues.

### Decoding error

We quantified the network’s memory performance by decoding the stimulus at the end of the delay. Because we used a deterministic simulation, we modeled the noise post-hoc using Poisson random number generator. We assume that the spike generation is random and independent across neurons. For each excitatory neuron indexed by θ, we multiplied its firing rate *r*_θ_(θ_0_) (in Hz), where θ_0_ denotes the true stimulus location, by 0.2 and used the product as the mean of the Poisson random number to model its spike count in 200ms. We denote this stochastic spike count as *n*_θ_(θ_0_). We then decoded the stimulated location ${\tilde{\theta }}_{0}$ from *n*_θ_(θ_0_) with a simple population-vector decoder [65]:

$${\tilde{\theta }}_{0}=\mathrm{angle}(\int_{-\pi }^{\pi }e^{i\theta }n_{\theta }(\theta_{0})d\theta )=\mathrm{atan}2(\int_{-\pi }^{\pi }\sin(\theta )n_{\theta }(\theta_{0})d\theta ,\int_{-\pi }^{\pi }\cos(\theta )n_{\theta }(\theta_{0})d\theta ).$$ (28)

The error is quantified by the cosine distance between the decoded location and true stimulus:

$$error(\theta_{0})=\langle 1-\cos(\theta_{0}-{\tilde{\theta }}_{0})\rangle .$$ (29)

At each trial, we freeze the network connectivity and simulate the response *r*_θ_(θ_0_) for each stimulus θ_0_. The random generation of spike counts was repeated 20 times and averaged for each θ_0_. We quantified the average error across all stimulus locations θ_0_. In directional statistics, averaging the cosine distance is a dispersion measure analogous to the total variation about a given angle [66]. After perturbation, when there is no spatial information at the end of the delay, ${\tilde{\theta }}_{0}$ would be uniformly distributed, and the average error would be one, while if the spatially patterned activity is persistent with no drift, the decoding error would be close to zero.

For convenience, at each trial we stimulated the network at all the preferred locations of the neurons, that is, at discrete locations. However, the network composed of finite neurons is able to encode continuous values in principle, and the decoded location can be between the preferred locations. When the network was stimulated at locations between the preferred locations and this continuous decoded location was used to quantify the error, the decoding error was not qualitatively different from those obtained using discrete locations (not shown).

### Spatial selectivity and translation-invariance

The spatial selectivity of each neuron was quantified by calculating the first Fourier component of its tuning curve given as

$$F1_{\theta }=\|\frac{1}{2\pi }\int_{-\pi }^{\pi }e^{i\theta_{0}}r_{\theta }(\theta_{0})d\theta_{0}\|,$$ (30)

where *r*_θ_(θ_0_) is the neuronal activity at the end of the delay period of a trial stimulated at θ_0_, where θ indicates the neuronal index as in Eq 26.

We calculated the mean and standard deviation across neurons.


$$\mathrm{mean}(F1)=\frac{1}{2\pi }\int_{-\pi }^{\pi }{F1}_{\theta }d\theta$$

$$\mathrm{td}(F1)=\sqrt{\frac{1}{2\pi }\int_{-\pi }^{\pi }{\left(F1_{\theta }-\mathrm{mean}(F1)\right)}^{2}d\theta }$$ (31)

The normalized std (std/mean) was used to quantify translation-invariance, as in [31].

## Supporting information

S1 Fig Related to Fig 4, Recovery dynamics dependence on learning parameters under homeostatic plasticity. A-F: Final *W*_*Exc*_/*W*_*inh*_ (top) and minimum number of trials for *W*_*Exc*_ to reach up to about 1% from perfect tuning (bottom) obtained by varying target rate *r*_*0*_ (A), learning speed α (B), perturbation strength *p* (C), *W*_*inh*_ (D), mean input strengths *c* (E), and by varying α and *r*_*0*_ together while α*r*_*0*_ is fixed (F). Final *W*_*Exc*_/*W*_*inh*_ was obtained by taking the mean (red curve) and standard deviation (shaded area) over 500 trials after reaching to the steady state. The final *W*_*Exc*_/*W*_*inh*_ is affected by *r*_*0*_, *W*_*inh*_, and *c* (A,D,E). Note that the effect of *W*_*inh*_ or *c* (D,E) can be reproduced by varying α and *r*_*0*_ together as derived analytically (F; Methods). On the other hand, varying α alone (B) or *p* (C) only affect the recovery speed in the opposite direction. *r*_*0*_ = 50, α = 4×10^−8^, *W*_*inh*_ = *c* = 500 unless otherwise specified and α*r*_*0*_ = 2×10^−6^ in F. Note different scales in Figs 4B and S1B where the horizontal axis in Fig 4B is in log scale to show a larger parameter range and that in S1B Fig here is in linear scale to be consistent with other panels.
(PDF)

S2 Fig Related to Fig 6, Elongation of time constant associated with each eigenvector similar to Fourier modes under differential plasticity. A: Time scale of each Fourier mode. For each Fourier mode, a time constant was estimated by projecting population activity onto a sinusoid of different frequencies (Methods) and fitting the time course with exponential decay. The negative reciprocals of these time constants have good correspondence with the eigenvalues shown in Fig 6D except for around the first 250 trials when the network transiently deviates from translation-invariance. B: Eigenvectors related to eigenvalues in Fig 6D during the evolution of learning dynamics. The real part of the eigenvectors corresponding to the first, third, and fifth leading eigenvalues is plotted (even ones omitted because of redundancy). After around 250 trials, the shape of the eigenvectors is close to sinusoids, suggesting restoration and maintenance of translation-invariance.
(PDF)

S3 Fig Related to Fig 7. Effects of changing learning speed and global perturbation strengths on recovered activity pattern under differential plasticity. Each panel is a snapshot of activity at the end of the delay period as in Fig 6B. The decoding error and spatial selectivity variability in Fig 7E and 7F were derived from these patterns. Note the color range twice as large as those in the main figures.
(PDF)

S4 Fig Related to Fig 8. Sensitivity of homeostatic plasticity on learning parameters in a spatially structured network. A-C: Effect of lower (A), higher target rates (B), and fast speed (C) under homeostatic plasticity. Top, middle and bottom rows show postsynaptic E-I ratio, activity pattern in three successive trials and the amplitude of peak activity, respectively, except for the bottom row of the third column. The postsynaptic E-I ratio of different neurons were shown in different colors (top). For lower target rate, activity decays and spatial information is lost (A). In contrast, for a higher target rate, the spatial pattern is maintained as well as the spatial information although the activity drift upwards (B). For fast homeostatic plasticity, the spatial locations were decoded using a population vector analysis as in Fig 6C, but for the entire delay period (C, bottom). Dashed lines are the stimulated locations. The parameters are *r*_0_ = 10 (A), *r*_0_ = 30 (B), *r*_0_ = 20 (C) and α_*h*_ = 10^−8^ (A,B), α_*h*_ = 10^−6^ (C).
(PDF)

S5 Fig Related to Fig 9. Effects of changing learning speed and postsynaptic perturbation strengths under differential plasticity. A-C: Decoding error (A), spatial selectivity variability (B) and activity pattern (C) recovered by differential plasticity with various learning rates after various level of postsynaptic perturbation. Note the color range of activity pattern (C) twice as large as those in the main figures.
(PDF)

S6 Fig Related to Fig 10. Effects of changing learning speed and presynaptic perturbation strengths under differential plasticity. A-C: Decoding error(A), spatial selectivity variability (B) and activity pattern (C) recovered by differential plasticity with various learning rates after various level of presynaptic perturbation. Note the color range of activity pattern (C) twice as large as those in the main figures.
(PDF)

S7 Fig Related to Fig 11. Effects of changing learning speed of combined plasticity under global perturbations. A-B: Spatial selectivity variability (A) and activity pattern (B) recovered by combined plasticity with various learning rates after global perturbation. Note the color range of activity pattern (B) twice as large as those in the main figures.
(PDF)

S8 Fig Related to Fig 11. Effects of changing learning speed of combined plasticity under postsynaptic perturbations. A-B: Spatial selectivity variability (A) and activity pattern (B) recovered by combined plasticity with various learning rates after postsynaptic perturbation. Note the color range of activity pattern (B) twice as large as those in the main figures.
(PDF)

S9 Fig Related to Fig 11. Effects of changing learning speed of combined plasticity under presynaptic perturbations. A-B: Spatial selectivity variability (A) and activity pattern (B) recovered by combined plasticity with various learning rates after presynaptic perturbation. Note the color range of activity pattern (B) twice as large as those in the main figures.
(PDF)

S10 FigRecovery of rate-coded persistent activity through differential plasticity that is always on but saturates for large derivatives.A-B: Time course of activity in a homogeneous population in successive trials (A) and phase-plane of activity and synaptic strength of recurrent excitation (B). Here we modified the plasticity rule such that $\frac{dw_{ij}}{dt}=-\alpha_{d}K\mathrm{sign}(\frac{dr_{i}}{dt})r_{j}$ for $|\frac{dr_{i}}{dt}|>K$ where sign(x) returns the sign of x and K gives the maximum amplitude of derivative that can be sensed by the learning mechanism. Unlike the horizontal jump in the phase plane where the plasticity is off during the stimulus presentation (Fig 2A), the red trajectory goes slightly downwards, showing “unlearning.” C-D: Activities with 10% perturbation (C) and after the recovery (D). K is set to be 1 activity unit/time unit. The stimulus period and mean input strengths are 10-time units and 10000, which are 5 times shorter and 10 times larger than those used Fig 1C–1F with the same rest of the parameters, such that the activity changes much faster in the stimulus period than in the delay period.(PDF)

S11 Fig Recovery of location-coded persistent activity through differential plasticity that is always on but saturates for large derivatives. A-B: Recovery of persistent activity in the spatially structured networks under the modified differential plasticity that is always on as in S10 Fig. C: Decrease of decoding error (black) and preservation of translation-invariance (red) with learning. D: Decoding error and normalized deviation of spatial selectivity for three different levels of perturbation. A-C shows the case of 10% perturbation in the E-to-E connection, and D shows 10, 20, and 30% perturbation. K is set to be 30 activity unit/s, and stimulus period and external input strength are 50 ms and 2025, respectively, which are 10 times shorter and 7.5 times larger than those used in Figs 6 and 7. The delay period is also shortened to 1s for faster simulation, while the rest of the parameters is the same as in Figs 6 and 7.
(PDF)
