# Supplementary figures and images for: Unsupervised learning for robust working memory
Source: PLoS Comput Biol. 2022 May 2;18(5):e1009083. doi: 10.1371/journal.pcbi.1009083 (PMC9098088; doi:10.1371/journal.pcbi.1009083)

Figure S1 - related to Figure 4

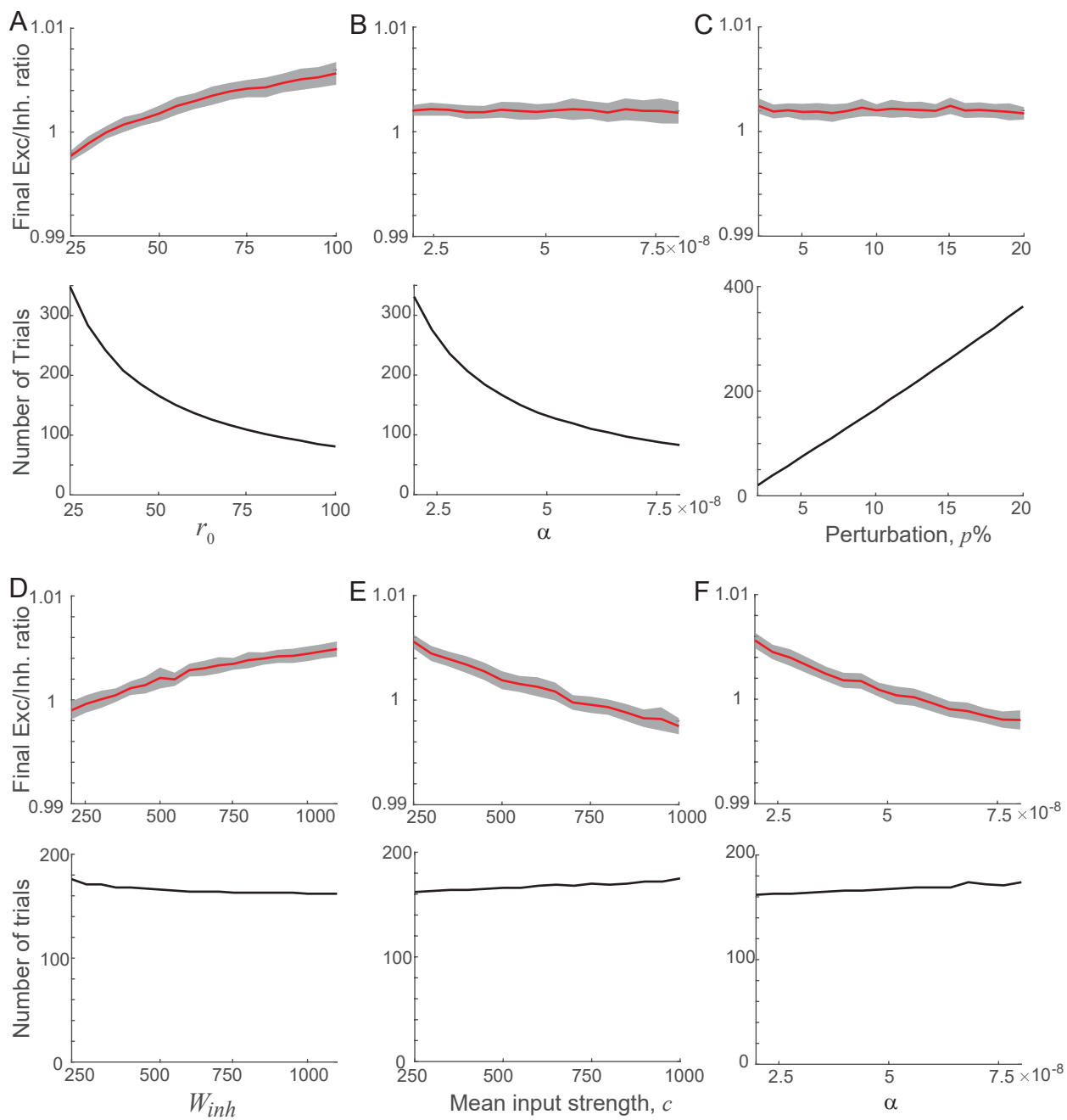

Supplement: S1 Fig — A-F: Final WExc/Winh (top) and minimum number of trials for WExc to reach up to about 1% from perfect tuning (bottom) obtained by varying target rate r0 (A), learning speed α (B), perturbation strength p (C), Winh (D), mean input strengths c (E), and by varying α and r0 together while αr0 is fixed (F). Final WExc/Winh was obtained by taking the mean (red curve) and standard deviation (shaded area) over 500 trials after reaching to the steady state. The final WExc/Winh is affected by r0, Winh, and c (A,D,E). Note that the effect of Winh or c (D,E) can be reproduced by varying α and r0 together as derived analytically (F; Methods). On the other hand, varying α alone (B) or p (C) only affect the recovery speed in the opposite direction. r0 = 50, α = 4×10^−8^, Winh = c = 500 unless otherwise specified and αr0 = 2×10^−6^ in F. Note different scales in Figs 4B and S1B where the horizontal axis in Fig 4B is in log scale to show a larger parameter range and that in S1B Fig here is in linear scale to be consistent with other panels. (PDF) [file pcbi.1009083.s001.pdf]

Figure S2 - related to Figure 6

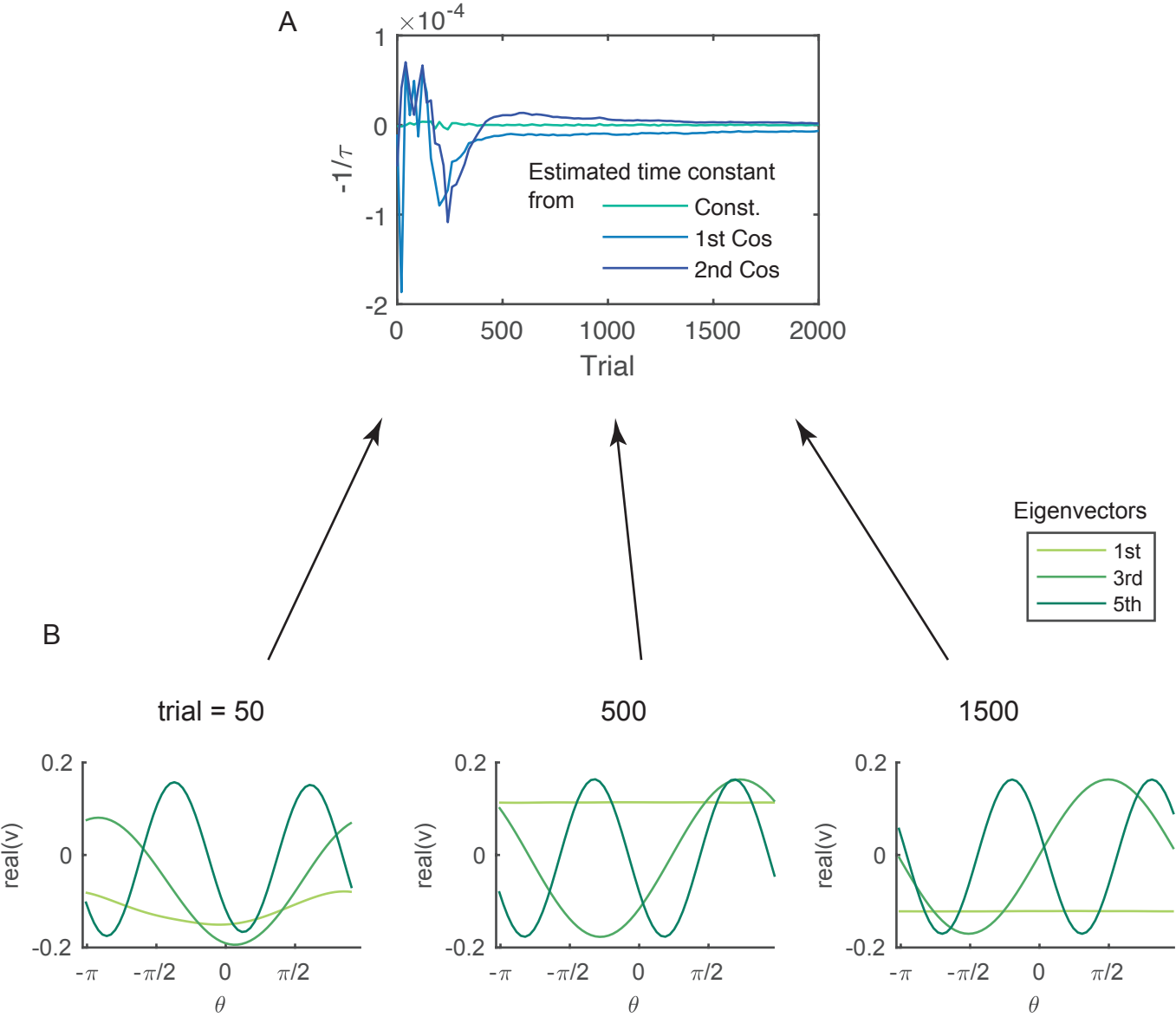

Supplement: S2 Fig — A: Time scale of each Fourier mode. For each Fourier mode, a time constant was estimated by projecting population activity onto a sinusoid of different frequencies (Methods) and fitting the time course with exponential decay. The negative reciprocals of these time constants have good correspondence with the eigenvalues shown in Fig 6D except for around the first 250 trials when the network transiently deviates from translation-invariance. B: Eigenvectors related to eigenvalues in Fig 6D during the evolution of learning dynamics. The real part of the eigenvectors corresponding to the first, third, and fifth leading eigenvalues is plotted (even ones omitted because of redundancy). After around 250 trials, the shape of the eigenvectors is close to sinusoids, suggesting restoration and maintenance of translation-invariance. (PDF) [file pcbi.1009083.s002.pdf]

Figure S3 - related to Figure 7

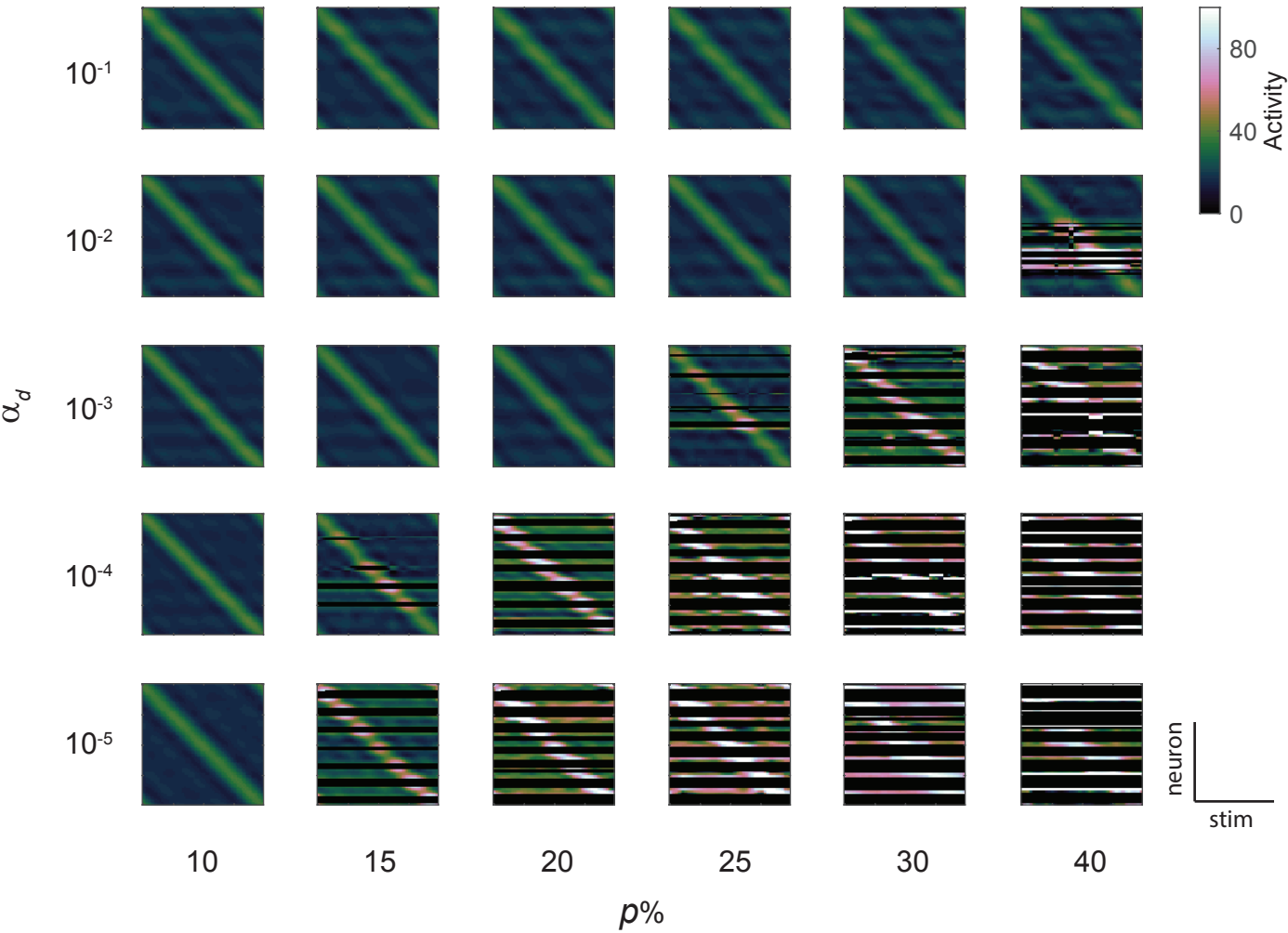

Supplement: S3 Fig — Effects of changing learning speed and global perturbation strengths on recovered activity pattern under differential plasticity. Each panel is a snapshot of activity at the end of the delay period as in Fig 6B. The decoding error and spatial selectivity variability in Fig 7E and 7F were derived from these patterns. Note the color range twice as large as those in the main figures. (PDF) [file pcbi.1009083.s003.pdf]

Figure S4 , related to Fig. 8

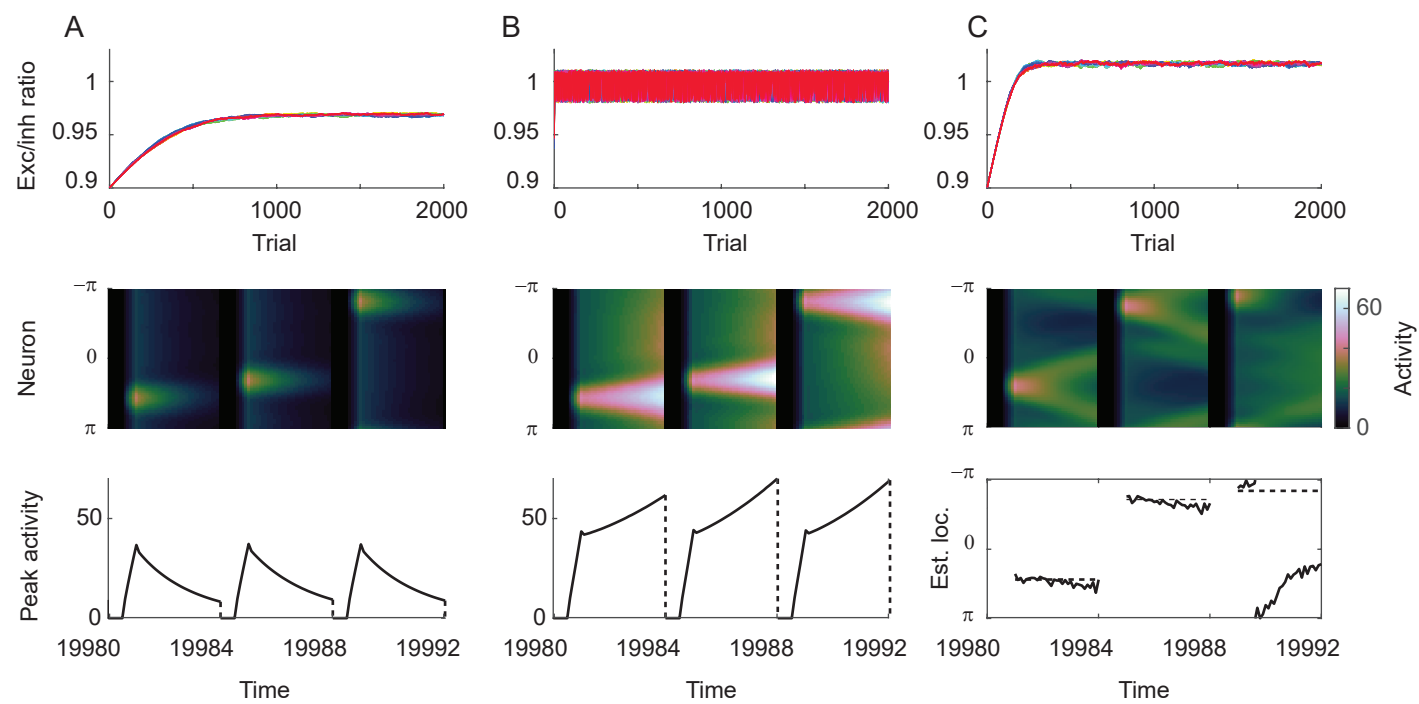

Supplement: S4 Fig — Sensitivity of homeostatic plasticity on learning parameters in a spatially structured network. A-C: Effect of lower (A), higher target rates (B), and fast speed (C) under homeostatic plasticity. Top, middle and bottom rows show postsynaptic E-I ratio, activity pattern in three successive trials and the amplitude of peak activity, respectively, except for the bottom row of the third column. The postsynaptic E-I ratio of different neurons were shown in different colors (top). For lower target rate, activity decays and spatial information is lost (A). In contrast, for a higher target rate, the spatial pattern is maintained as well as the spatial information although the activity drift upwards (B). For fast homeostatic plasticity, the spatial locations were decoded using a population vector analysis as in Fig 6C, but for the entire delay period (C, bottom). Dashed lines are the stimulated locations. The parameters are r0 = 10 (A), r0 = 30 (B), r0 = 20 (C) and αh = 10−8 (A,B), αh = 10−6 (C). (PDF) [file pcbi.1009083.s004.pdf]

Figure S5 - related to Figure 9

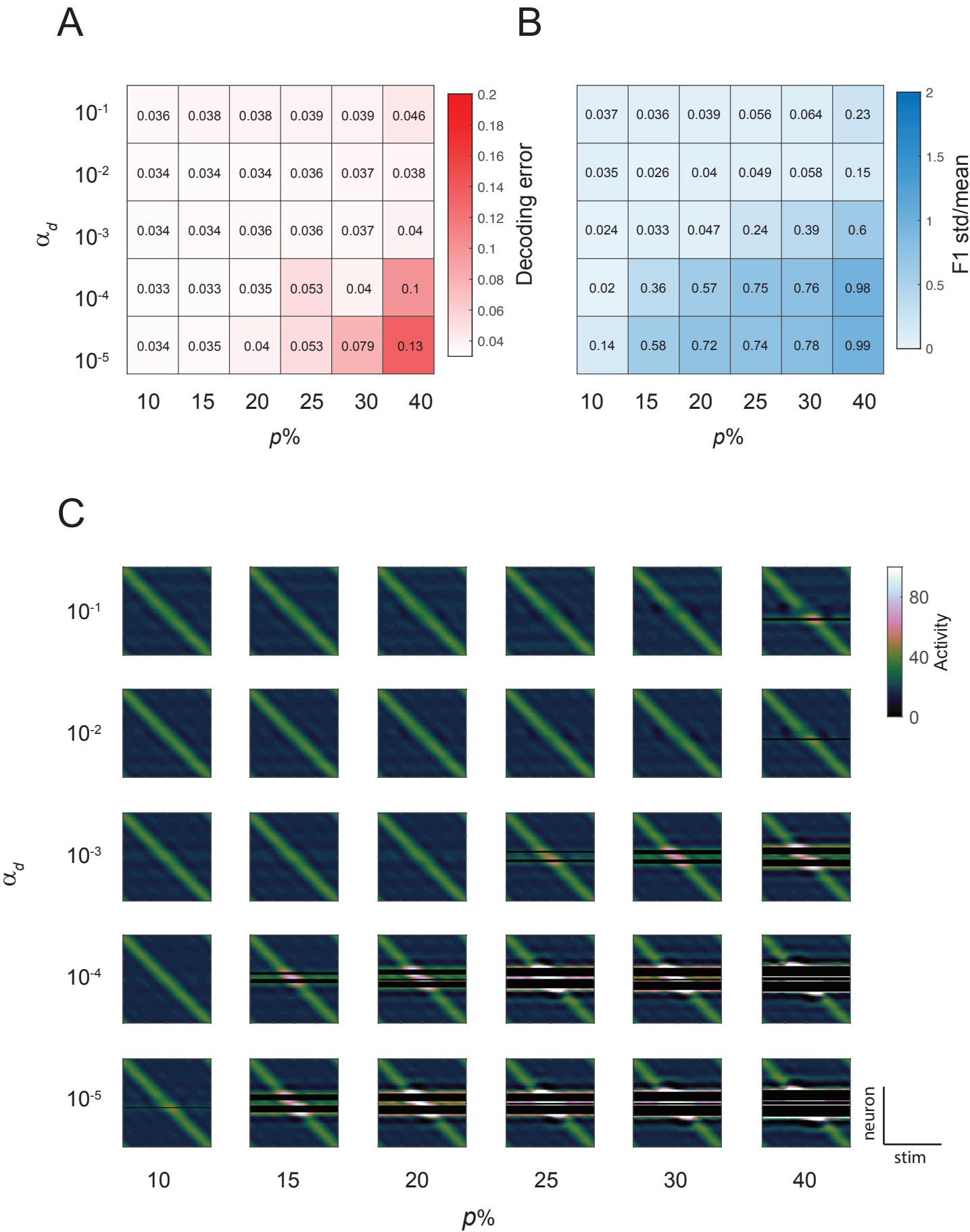

Supplement: S5 Fig — Effects of changing learning speed and postsynaptic perturbation strengths under differential plasticity. A-C: Decoding error (A), spatial selectivity variability (B) and activity pattern (C) recovered by differential plasticity with various learning rates after various level of postsynaptic perturbation. Note the color range of activity pattern (C) twice as large as those in the main figures. (PDF) [file pcbi.1009083.s005.pdf]

Figure S6 - related to Figure 10

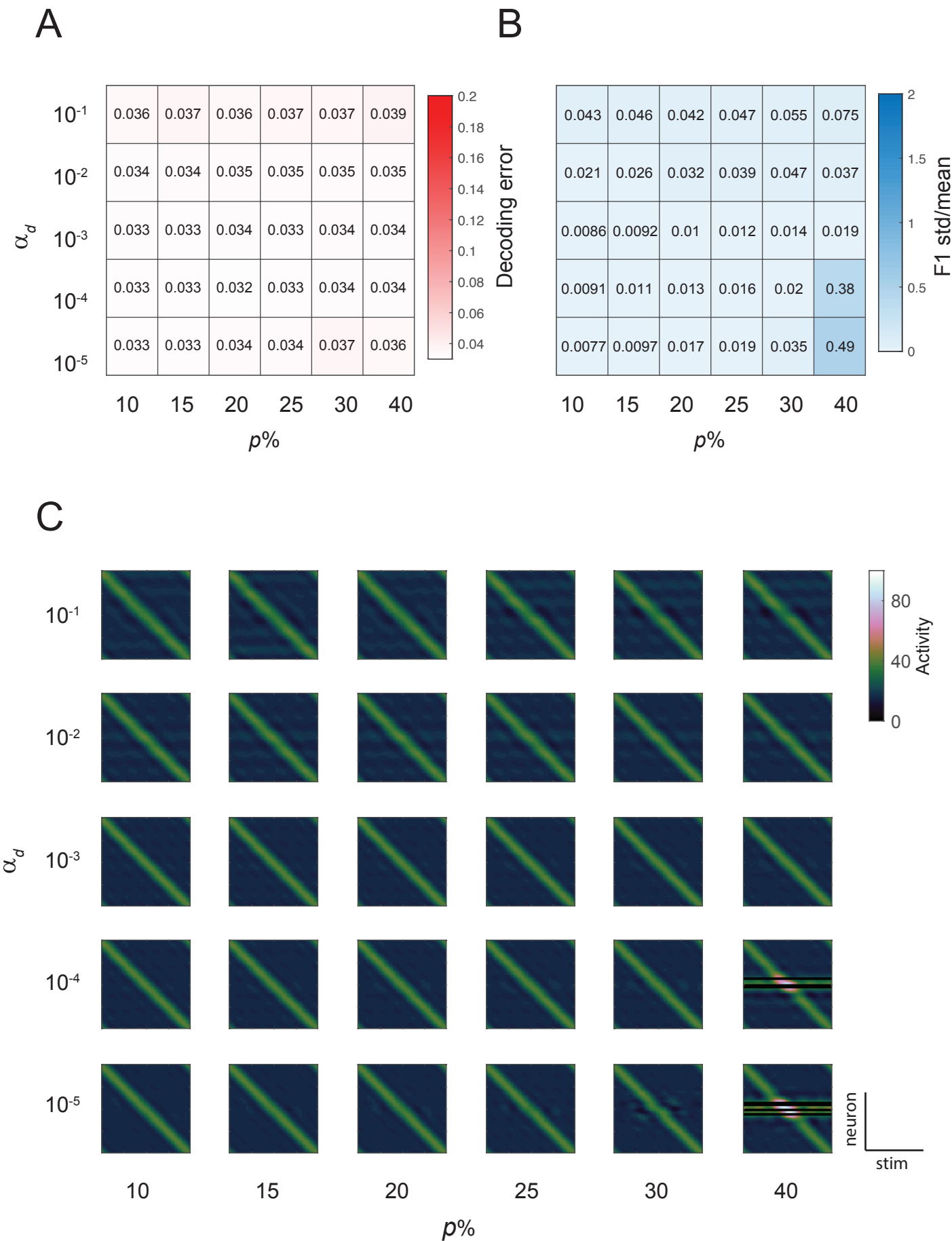

Supplement: S6 Fig — Effects of changing learning speed and presynaptic perturbation strengths under differential plasticity. A-C: Decoding error(A), spatial selectivity variability (B) and activity pattern (C) recovered by differential plasticity with various learning rates after various level of presynaptic perturbation. Note the color range of activity pattern (C) twice as large as those in the main figures. (PDF) [file pcbi.1009083.s006.pdf]

Figure S7 - related to Figure 11 Global perturbation

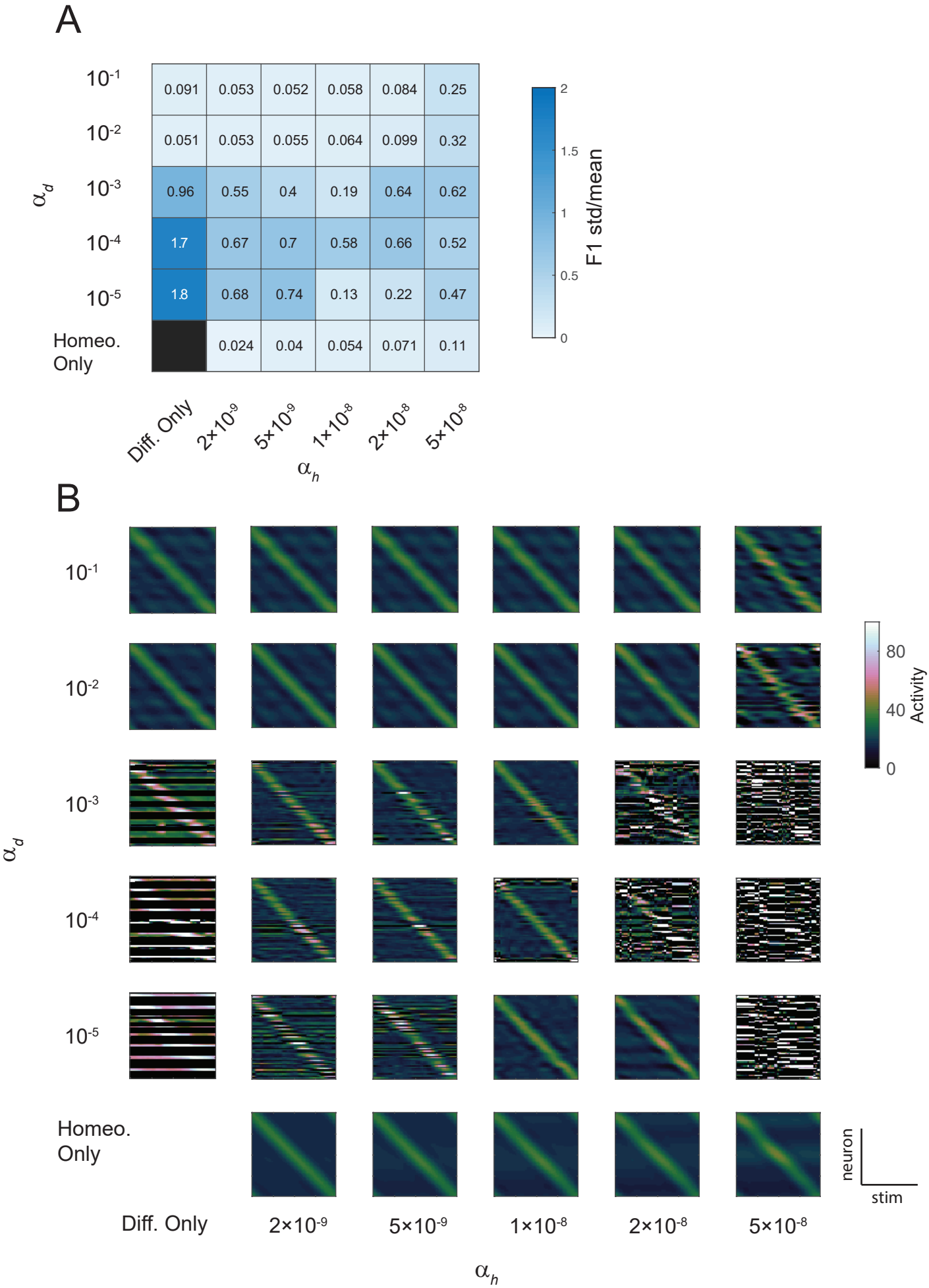

Supplement: S7 Fig — Effects of changing learning speed of combined plasticity under global perturbations. A-B: Spatial selectivity variability (A) and activity pattern (B) recovered by combined plasticity with various learning rates after global perturbation. Note the color range of activity pattern (B) twice as large as those in the main figures. (PDF) [file pcbi.1009083.s007.pdf]

Figure S8 - related to Figure 11 Postsynaptic perturbation

A

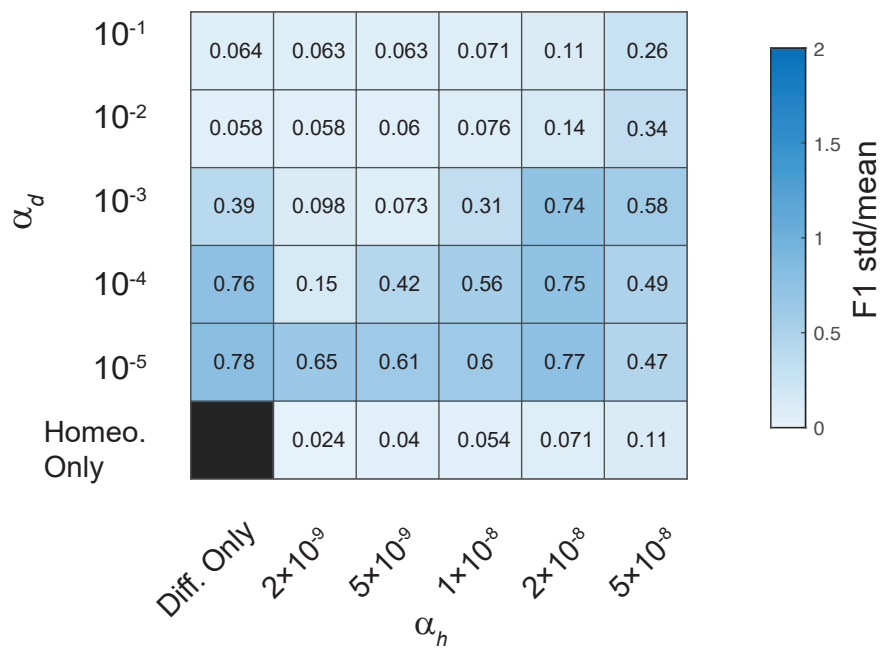

**B**

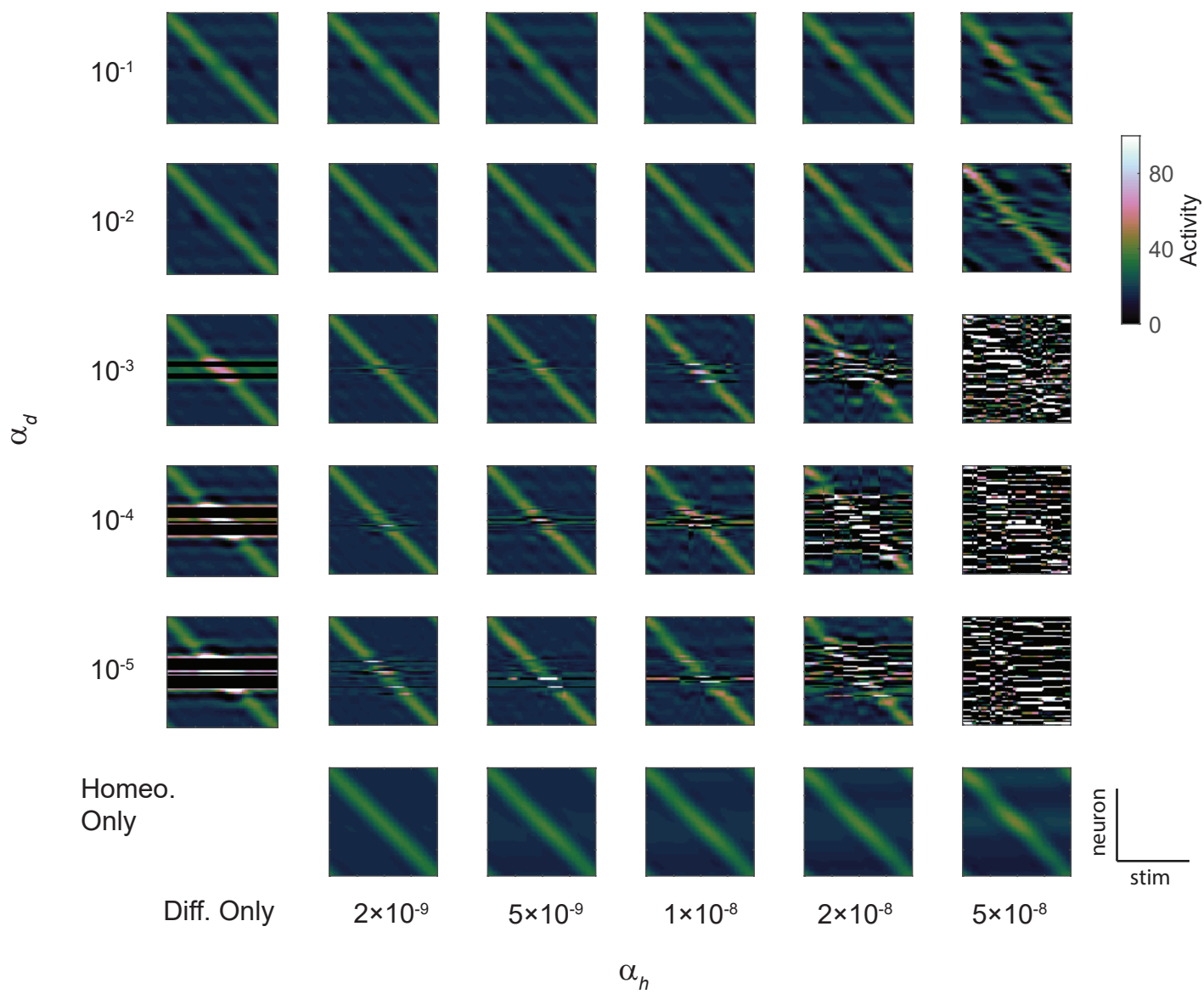

Supplement: S8 Fig — Effects of changing learning speed of combined plasticity under postsynaptic perturbations. A-B: Spatial selectivity variability (A) and activity pattern (B) recovered by combined plasticity with various learning rates after postsynaptic perturbation. Note the color range of activity pattern (B) twice as large as those in the main figures. (PDF) [file pcbi.1009083.s008.pdf]

Figure S9 - related to Figure 11 Presynaptic perturbation

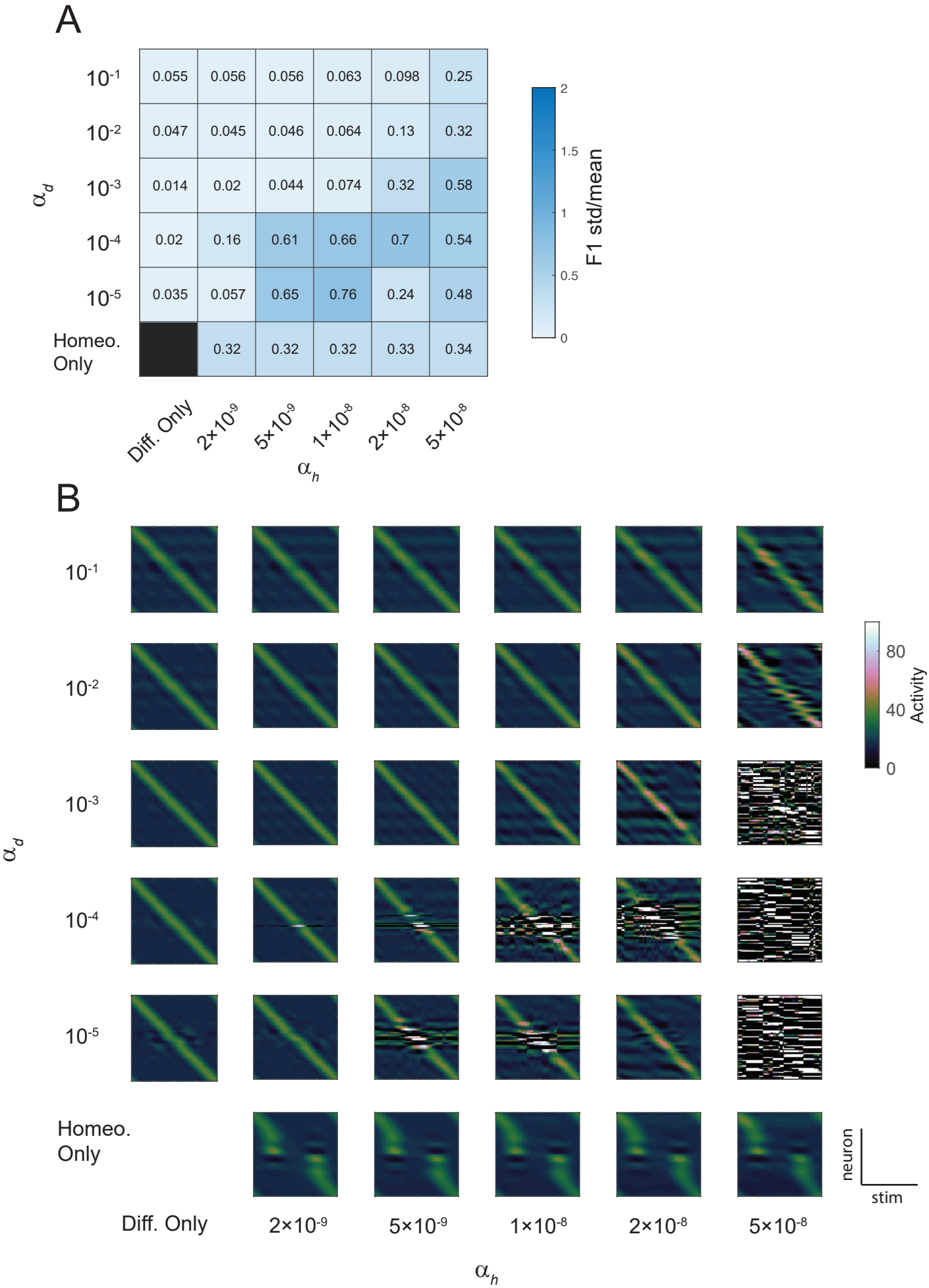

Supplement: S9 Fig — Effects of changing learning speed of combined plasticity under presynaptic perturbations. A-B: Spatial selectivity variability (A) and activity pattern (B) recovered by combined plasticity with various learning rates after presynaptic perturbation. Note the color range of activity pattern (B) twice as large as those in the main figures. (PDF) [file pcbi.1009083.s009.pdf]

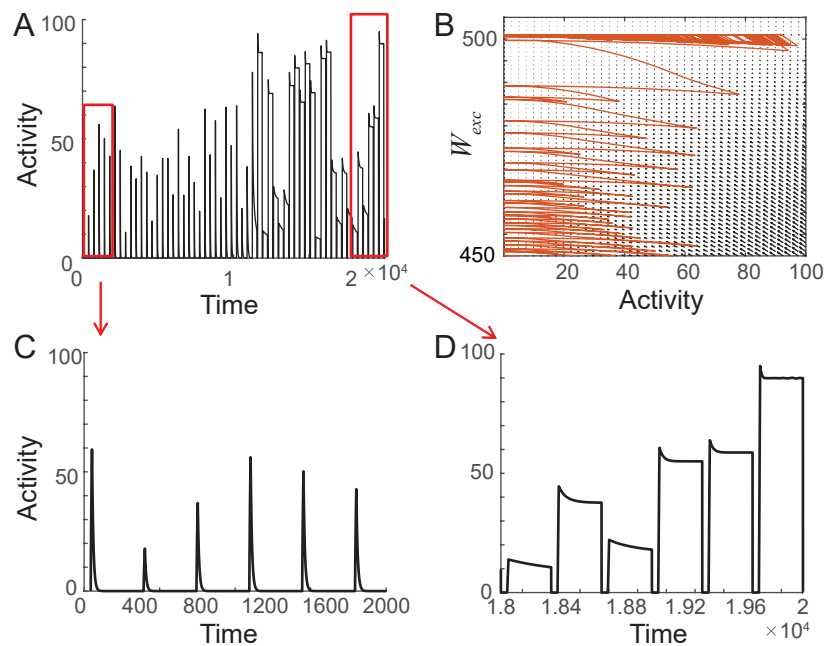

Supplement: S10 Fig — A-B: Time course of activity in a homogeneous population in successive trials (A) and phase-plane of activity and synaptic strength of recurrent excitation (B). Here we modified the plasticity rule such that dwijdt=−αdKsign(dridt)rj for |dridt|>K where sign(x) returns the sign of x and K gives the maximum amplitude of derivative that can be sensed by the learning mechanism. Unlike the horizontal jump in the phase plane where the plasticity is off during the stimulus presentation (Fig 2A), the red trajectory goes slightly downwards, showing “unlearning.” C-D: Activities with 10% perturbation (C) and after the recovery (D). K is set to be 1 activity unit/time unit. The stimulus period and mean input strengths are 10-time units and 10000, which are 5 times shorter and 10 times larger than those used Fig 1C–1F with the same rest of the parameters, such that the activity changes much faster in the stimulus period than in the delay period. (PDF) [file pcbi.1009083.s010.pdf]

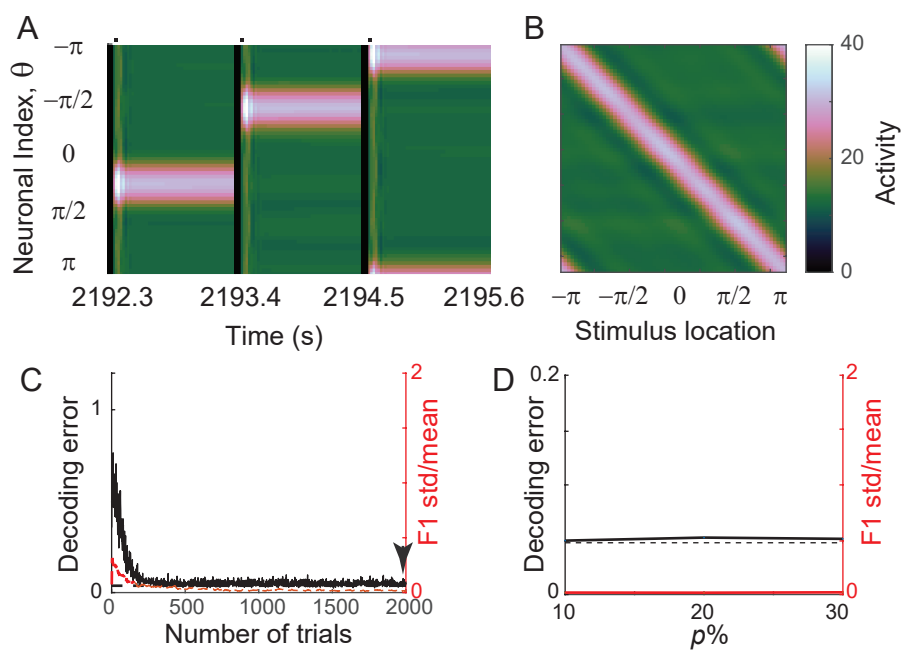

Supplement: S11 Fig — A-B: Recovery of persistent activity in the spatially structured networks under the modified differential plasticity that is always on as in S10 Fig. C: Decrease of decoding error (black) and preservation of translation-invariance (red) with learning. D: Decoding error and normalized deviation of spatial selectivity for three different levels of perturbation. A-C shows the case of 10% perturbation in the E-to-E connection, and D shows 10, 20, and 30% perturbation. K is set to be 30 activity unit/s, and stimulus period and external input strength are 50 ms and 2025, respectively, which are 10 times shorter and 7.5 times larger than those used in Figs 6 and 7. The delay period is also shortened to 1s for faster simulation, while the rest of the parameters is the same as in Figs 6 and 7. (PDF) [file pcbi.1009083.s011.pdf]
